# Bringing translational structure to complex immune-mediated conditions: the ACT (Activation–Constraint–Tissue Context) framework for divergent phenotypes

**DOI:** 10.3389/fimmu.2026.1866922

**Published:** 2026-10-23

**Authors:** Jeffrey John Lorsung

**Affiliations:** 1 Accenture, New York, NY, United States; 2 Independent Researcher, Sarasota, FL, United States

**Keywords:** clinical immunology, dimensionality reduction (DR), divergent immune phenotypes, immune-state trajectories, longitudinal immune profiling, state-space modeling, systems immunology, translational immunology

## Abstract

Immune-mediated outcomes diverge across individuals with similar genetic backgrounds and environmental exposures, reflecting trajectories through a dynamic, context-dependent, and partially stochastic state space rather than fixed molecular endpoints. Despite advances in systems immunology, computational modeling, and multi-omic profiling, translating this complexity into clinically actionable reasoning remains difficult: these disciplines generate increasingly detailed representations of immune behavior that deepen mechanistic understanding while raising barriers to integration across clinical, experimental, and computational domains. A clinician at the bedside cannot reason across tens of thousands of molecular variables, cell states, pathways, and contextual modifiers simultaneously. We adopt a reduction-oriented perspective — not because immune data are insufficient, but because high-dimensional biological information must be organized into a reduced representational space before it can support cross-domain reasoning. When a system is too complex to reason about directly, progress may depend on identifying a minimal set of variables that preserves explanatory power while enabling tractable inference. Rather than modeling the immune system in full mechanistic detail, we seek a constrained representation capturing the minimal structure necessary to characterize clinically observable immune phenotypes, consistent with principles from state-space modeling, dimensionality reduction, and systems biology. Here, we introduce ACT — Activation, Constraint, and Tissue Context — a reduced-dimensional immune phenotype space for reasoning about immune state across systemic and tissue-localized compartments. Within ACT, clinical observations, laboratory measurements, and multi-omic data are interpreted as projections of a latent immune state that evolves over time under environmental influence, host predisposition, therapeutic intervention, tissue context, and stochastic perturbation. Immune phenotype is thus represented not as a static label, but as a trajectory through reduced immune-state space. ACT does not infer molecular mechanism directly from phenotypic or reduced-state data. Instead, it provides a structured mapping from data to state, state to trajectory, and trajectory to pathway-level hypothesis generation — linking parsimonious state representation with trajectory-based reasoning to offer a conceptual scaffold for translating between clinical observation, multi-omic data, and pathway-level hypothesis generation in complex immune-mediated conditions. The goal is not to replace high-resolution molecular data, systems immunology, or mechanistic modeling, but to provide a common translational coordinate system for reasoning about immune phenotype across scales of observation and disciplinary boundaries — supporting communication, interpretation, and hypothesis generation across clinical, experimental, and computational domains.

## 1 Introduction

Disease may be best understood not as a static molecular endpoint, but as a trajectory through a partially observable, dynamic immune state space — shaped by deterministic biological process, environmental exposure, host predisposition, therapeutic intervention, tissue context, and stochastic perturbation. Immune-mediated outcomes often diverge sharply across individuals with similar genetic backgrounds and environmental exposures, including within families: related individuals may develop infection susceptibility, autoimmunity, inflammatory disease, or clonal lymphoid disorders despite overlapping exposures and partial genetic commonality.

This divergence is not an abstraction. A patient living through decades of autoimmune phenomena does not experience a series of disconnected diagnoses — they experience a trajectory, with some underlying cause threading through it. A caregiver, watching across a family and across years, often notices shifts in a relative’s disease state long before those shifts are captured by any single clinical measurement. Yet much of clinical medicine remains organized around molecular snapshots, evaluated within diagnosis- or pathway-specific specialties. That specialization is essential to good care, but it also means immune phenotype is typically treated as an endpoint to be classified rather than a state to be tracked — leaving patients whose illness crosses clinical domains without a shared language for what clinicians, caregivers, and patients are each independently observing.

Trajectory-based reasoning is not new. Oncology already reasons this way at the bedside — a tumor is staged not only by what it is but by whether it is stable, progressing, or in remission. Systems biology and computational immunology have gone further, building explicit trajectory models at the molecular level: pseudotime analysis, lineage reconstruction, cell-state transition modeling, spatial omics, and dynamical modeling of transcriptional and signaling programs now routinely represent biological transitions as paths through high-dimensional state spaces (1, 2), advancing understanding of immune activation, differentiation, tissue remodeling, tumor evolution, and disease progression (2, 3). But these approaches operate at the level of cells, genes, transcripts, or molecular programs. They do not, on their own, provide a reduced, clinically interpretable coordinate system in which bedside observation, laboratory data, multi-omic measurement, family-level divergence, and pathway-level hypotheses can all be reasoned about within a common structure.

Recent systems immunology has increasingly named this gap. High-dimensional immune profiling, multi-omic integration, AI-assisted modeling, network pharmacology, and quantitative systems pharmacology continue to reveal the complexity of immune behavior while generating ever more complex data (4, 5), but greater molecular resolution does not, by itself, produce clinically actionable interpretation (4, 5). The open question is no longer whether immune biology can be measured in finer detail, but how that information can be organized into a representation that supports reasoning across clinical, experimental, and computational domains at once (4, 5).

Work on inborn errors of immunity poses a complementary challenge from the opposite direction. Human genetic studies have shown that highly specific perturbations in immune pathways can produce selective vulnerability to infection, autoimmunity, immune dysregulation, and markedly variable clinical expression (6, 7), with incomplete penetrance, modifier effects, and tissue context further shaping outcomes such that shared genetic predisposition does not guarantee shared phenotype (8). This has transformed pathway-level reasoning in clinical immunology, but pathway-specific discovery does not by itself yield a general framework for comparing divergent trajectories across individuals, families, tissues, and disease domains.

Across both directions — molecular trajectory modeling reaching for clinical relevance, and pathway discovery reaching for generality — the same structure is missing: a translational coordinate system through which observable phenotype, longitudinal immune behavior, and pathway-level hypothesis can be related. What these observations share is that immune phenotype reflects the integrated behavior of a dynamic system, not the isolated output of a single pathway or defect.

We therefore represent the underlying immune state as a latent process evolving under both deterministic and stochastic influence — the general logic of state-space models, in which latent dynamics evolve through time while observable measurements offer only partial, noisy access to the underlying state (9, 10) expressed in Itô style formulation:


$${\text{dS}}_{\text{t}}=\text{f}\left({\text{S}}_{\text{t}},{\text{ u}}_{\text{t}},\text{ θ},\text{ t}\right)\text{dt}+\Sigma \left({\text{S}}_{\text{t}},{\text{ u}}_{\text{t}},\text{ θ},\text{ t}\right){\text{dW}}_{\text{t}}$$

Here, S_t_ denotes the latent immune state at time t; f(·) represents deterministic drift induced by physiological regulation, disease processes, host predisposition, environmental exposure, and therapeutic intervention; Σ(·) represents state-dependent stochastic influence; and W_t_ denotes a Wiener process. The parameter vector θ represents relatively metastable host features — genetic predisposition and baseline regulatory architecture — that remain stable under ordinary perturbation but may shift following sufficient cumulative or threshold-level change, while u_t_ represents time-varying external influences including infection, vaccination, medication, tissue injury, microbiome shifts, malignancy, and other environmental exposures.

Observable clinical, laboratory, imaging, and molecular measurements are treated as noisy projections of this latent state:


$${\text{Y}}_{\text{t}}=\text{h}\left({\text{S}}_{\text{t}},{\text{ u}}_{\text{t}},\text{ θ},\text{ t}\right)+{\text{ϵ}}_{\text{t}}$$

Here, Y_t_ denotes observed measurements, h(·) links latent state and contextual inputs to observable features, and ϵ_t_ represents measurement error and unmodeled variability. Similar distinctions between latent dynamics and noisy observation underpin state-space approaches across biological and dynamical systems (9, 10).

Although conceptually straightforward, this formulation presents a practical problem. The full latent state S_t_ is high-dimensional, only partially observable, and not directly usable for clinical reasoning — a clinician cannot reason simultaneously across tens of thousands of molecular variables, cell states, pathways, and contextual modifiers. Yet many clinically meaningful observations can be interpreted in terms of a smaller number of aggregate system properties. We therefore seek a reduced representation that preserves clinically relevant information while supporting tractable reasoning.

We define this reduction through a mapping φ from the full latent state to a clinically interpretable immune phenotype coordinate:


$${\text{Z}}_{\text{t}}=\text{φ}\left({\text{S}}_{\text{t}}\right)=\left({\text{A}}_{\text{t}},{\text{ C}}_{\text{t}},{\text{ T}}_{\text{t}}\right)$$

where A_t_ denotes immune activation, C_t_ denotes effective immune constraint or regulatory limitation, and T_t_ denotes tissue context. ACT operationalizes this reduction through three interpretable dimensions — Activation, Constraint, and Tissue Context — treated as compressed summaries of broader immune-state behavior rather than direct measurements of individual molecular mechanisms.

Tissue Context is not intended to imply that tissue is a single scalar dimension equivalent to activation or constraint; rather, it represents the systemic or tissue-localized compartment within which activation and constraint are expressed — consistent with findings from spatial immunology and spatial omics demonstrating that anatomical organization, stromal architecture, local immune niches, and tissue-specific cellular interactions shape immune behavior (11). Where greater resolution is required, ACT can be extended to compartment-specific representations, described in the following section.

The resulting ACT immune phenotype space is not proposed as a complete mechanistic model of immune biology, a new measurement platform, or a novel computational estimator. Rather, it is a reduced translational coordinate system for estimating, comparing, and reasoning about immune-state trajectories. Clinical observations, laboratory measurements, and multi-omic data are used to position an individual within this space over time, and the resulting trajectory can then be used to constrain and prioritize plausible pathway classes for downstream evaluation — a hypothesis-generation step rather than direct inference from the same molecular features used to construct the trajectory.

The primary contribution of this work is therefore not the invention of trajectory inference, state-space modeling, pseudotime analysis, or latent-state representations, all already established within computational biology and systems immunology (1–3, 9). Rather, it is the integration of clinically interpretable state reduction, longitudinal trajectory reasoning, and pathway-class prioritization into a common translational coordinate system for reasoning across individuals and families — particularly where shared predisposition and overlapping exposures give rise to divergent immune-mediated outcomes across infection susceptibility, autoimmunity, inflammatory disease, and clonal immune disorders.

## 2 The tri-axis immune state representation

The reduction map introduced above, Z_t_ = φ(S_t_) = (A_t_, C_t_, T_t_), is developed fully here. We adopt function-argument notation — Z(t) = (A(t), C(t), T(t)) — for the reduced ACT state and all subsequent trajectory analysis, consistent with its treatment as a discrete-time system in Section 6. Activation A(t) represents effective signal propagation from immune sensing to downstream response — cytokine signaling, interferon responses, inflammatory amplification, and effector activation. Constraint C(t) represents regulatory and selection processes that modulate, limit, resolve, or shape immune activity — tolerance mechanisms, checkpoint pathways, regulatory-cell function, contraction dynamics, and clonal selection. Tissue Context T(t) represents the systemic or tissue-localized compartment within which activation and constraint are expressed.

Immune phenotype can be understood as the balance and interaction among these three dimensions: shifts in activation, strengthening or breakdown of constraint, and changes in tissue localization collectively define observable immune behavior at a given timepoint. These axes are not direct molecular measurements or mechanistically independent variables — they are effective system properties, compressed summaries chosen for clinical interpretability, biological relevance, and computational compatibility. **Figure 1** illustrates this reduction: clinical observables and high-dimensional gene-expression data represent partial, low- and high-dimensional projections, respectively, of the same underlying latent immune state, with the inferred position in tri-axis space represented as a probability distribution reflecting estimation uncertainty.

**Figure 1:**
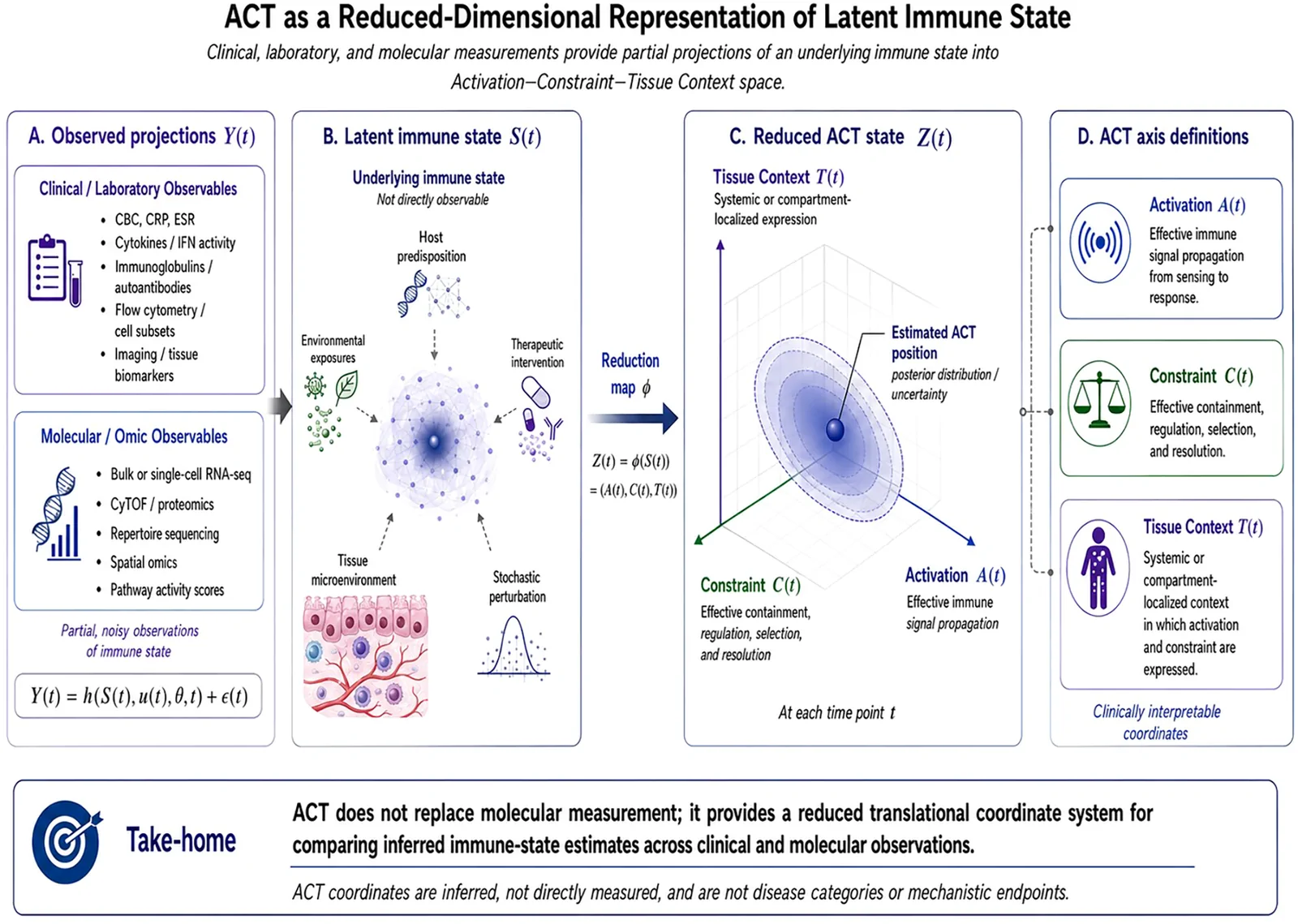
ACT as a reduced-dimensional representation of latent immune state. **(A)** Observed projections Y(t): clinical, laboratory, and molecular/omic measurements provide partial, noisy observations of immune state. **(B)** Latent immune state S(t), shaped by host predisposition, environmental exposures, therapeutic intervention, tissue microenvironment, and stochastic perturbation. **(C)** Reduced ACT state Z(t) = φ(S(t)), with the estimated position shown as a posterior distribution reflecting uncertainty. **(D)** Definitions of the Activation, Constraint, and Tissue Context axes.

### 2.1 Tissue Context at clinical and computational resolution

Tissue Context plays different roles depending on resolution. Clinically, it functions adequately as a single coordinate — a clinician distinguishing systemic from localized disease, or blood-based activation from mucosal inflammation, is already reasoning in terms of tissue context. Computational use demands more precision, since activation and constraint are always expressed within some anatomical or microenvironmental setting. Where multiple compartments are involved, ACT extends to a compartment-specific form:


$${\text{Z}}_{\text{k}}\left(\text{t}\right)=\left({\text{A}}_{\text{k}}\left(\text{t}\right),{\text{ C}}_{\text{k}}\left(\text{t}\right),{\text{ T}}_{\text{k}}\right)$$

where A_k_(t) and C_k_(t) denote activation and constraint local to compartment k, and T_k_ indexes the relevant context. This lets ACT represent divergent activation–constraint balances within the same individual — contained mucosal inflammation alongside systemic quiescence, or localized tumor-associated activation alongside systemic suppression — while preserving the same structure at each resolution. Tissue Context is thus best understood as a single clinical coordinate that resolves, under computational inspection, into a set of compartments in which activation and constraint are separately expressed — a question of representational scale, not contradiction. Critically, a compartment’s history of prior activation–constraint imbalance conditions its future vulnerability, so tissue context functions not only as a site of expression but as a record of accumulated trajectory history within that compartment.

### 2.2 Biological grounding

ACT reflects established biology in which activation and constraint are tightly coupled. The cGAS–STING pathway illustrates this: cytosolic DNA sensing activates STING-dependent interferon responses (12, 13), but persistent activation of the same pathway can drive inflammatory or autoimmune pathology, requiring regulatory mechanisms to limit its consequences (13). Tumor immunity shows the same relationship: immunogenic cell death and STING-dependent sensing of tumor DNA support anti-tumor immune recognition and type I interferon production in the tumor microenvironment (14, 15) — pathways sitting at the intersection of Activation and Constraint rather than belonging to either alone.

These examples also show why Tissue Context must be first-order: the consequence of a pathway like cGAS–STING depends on where it occurs and what local environment shapes the response. This is reinforced at atlas scale — Human Cell Atlas work has shown cell identity cannot be separated from anatomical context (16), and cross-tissue immune profiling has demonstrated tissue-specific transcriptional programs and clonal architecture even among related immune populations (17). B-cell and germinal-center biology reinforces the same point: differentiation, class switching, affinity maturation, and memory formation depend on local lymphoid architecture and tissue-specific cues, not activation magnitude alone (18).

### 2.3 From high-dimensional measurement to ACT state space

Clinical, laboratory, imaging, and molecular measurements can be understood as projections of the same underlying immune state at different resolutions — coarse clinical observables and high-dimensional assays (flow cytometry, CyTOF, transcriptomics, spatial omics, repertoire sequencing) alike. ACT treats all such observations as partial, noisy evidence for a position within reduced immune-state space, with different modalities informing different axes: cytokine and interferon-stimulated gene programs inform Activation; regulatory-cell phenotypes and autoantibody production inform Constraint; tissue biopsy, imaging, and spatial omics inform Tissue Context. Because these modalities differ in units, distributions, and directionality, observations are first converted into directionally meaningful abnormality signals, standardized relative to individual or population baseline, before being combined — preventing incompatible measurement types from being averaged as though equivalent. **Figure 2** summarizes this pipeline: high-dimensional measurements are passed through dimensionality-reduction and state-inference methods to yield a low-dimensional latent state estimate, expressed in tri-axis form as a position (with uncertainty) in ACT space.

**Figure 2:**
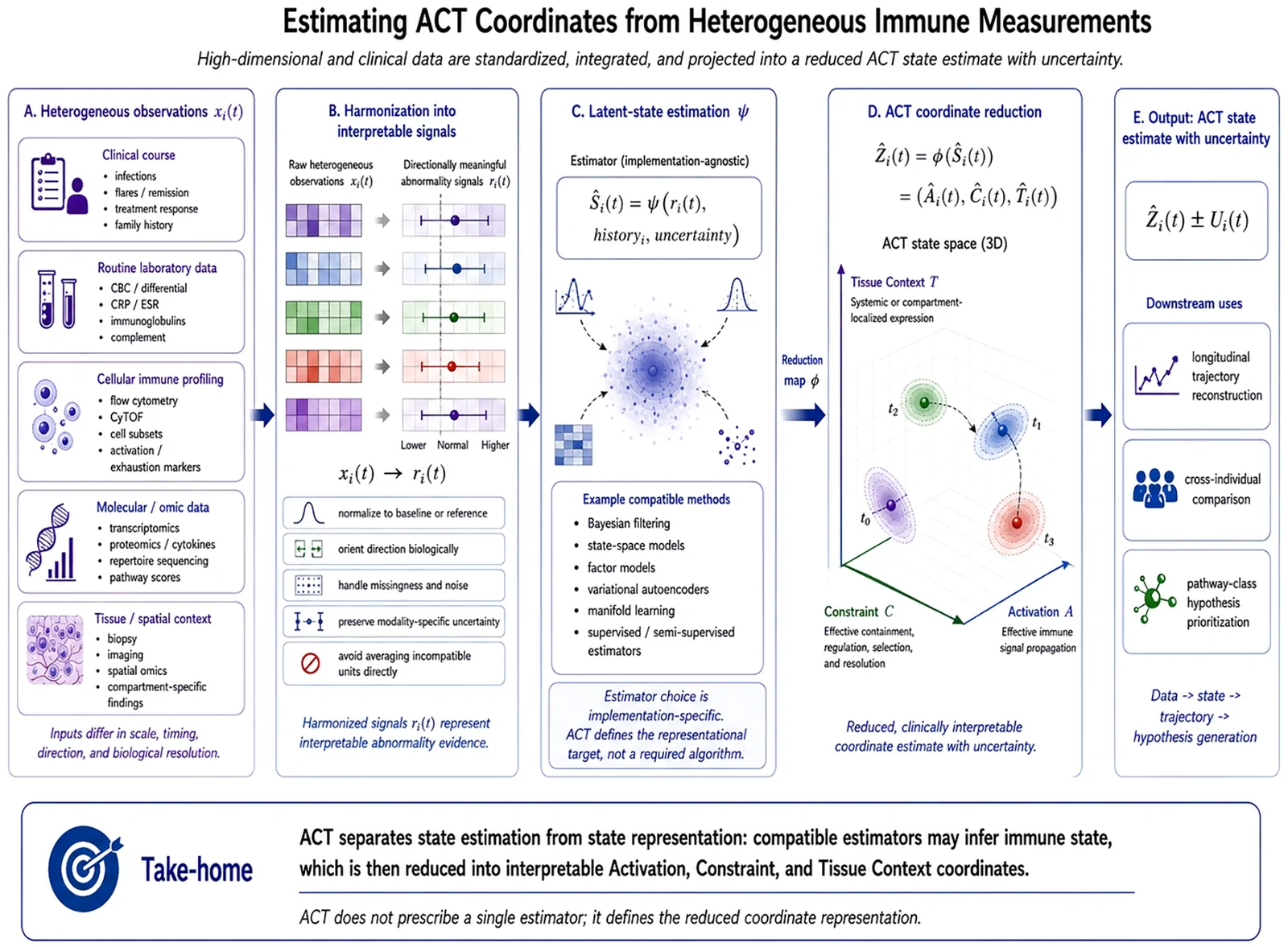
Mapping high-dimensional immune data into ACT state space. **(A)** Heterogeneous observations *x*_i_(*t*), spanning clinical course, routine laboratory data, cellular immune profiling, molecular/omic data, and tissue/spatial context. **(B)** Harmonization of raw observations into directionally meaningful abnormality signals *r*_i_(*t*). **(C)** Latent-state estimation ψ, which may use any compatible estimator. **(D)** Reduction map φ, yielding ACT coordinates in three-dimensional state space across successive time points. **(E)** Output: an ACT state estimate with uncertainty, and its downstream uses in trajectory reconstruction, cross-individual comparison, and pathway-class hypothesis prioritization.

Existing dimensionality-reduction and latent-variable methods — embedding, Bayesian filtering, state-space estimation — can be used to estimate an individual’s ACT position and its uncertainty from heterogeneous inputs. ACT treats the choice of estimator as a modeling decision, not its defining feature, separating how state is estimated from data from how that estimate is represented and compared over time — and addresses only the latter.

### 2.4 Family and cross-individual divergence

Because ACT places individuals in a shared coordinate system, related individuals with shared genetic predisposition can be understood as beginning from overlapping regions of immune-state space, with trajectories that diverge over time due to exposure, timing, tissue involvement, therapeutic intervention, and stochastic variation. One relative may move toward infection susceptibility from insufficient activation; another toward autoimmunity from persistent activation or constraint failure; a third toward clonal lymphoid pathology from selection failure in a specific tissue context. Viewed as isolated diagnoses these appear unrelated; viewed as trajectories through shared ACT space, they may represent divergent expressions of shared predisposition — particularly relevant given the incomplete penetrance and variable expressivity documented in inborn errors of immunity (6–8). ACT reframes the question from “do these individuals share a diagnosis?” to “do they share an underlying configuration that diverges through different trajectories?” **Figure 3** illustrates this schematically for a hypothetical family of five related individuals who begin in a shared region of ACT space and diverge over time into distinct outcome regions — autoimmune/autoinflammatory, healthy/controlled, infection-susceptible, clonal/lymphoid, and oscillatory/mixed — driven by differential exposures, internal perturbations, and stochastic variation rather than by differences present at initialization.

**Figure 3:**
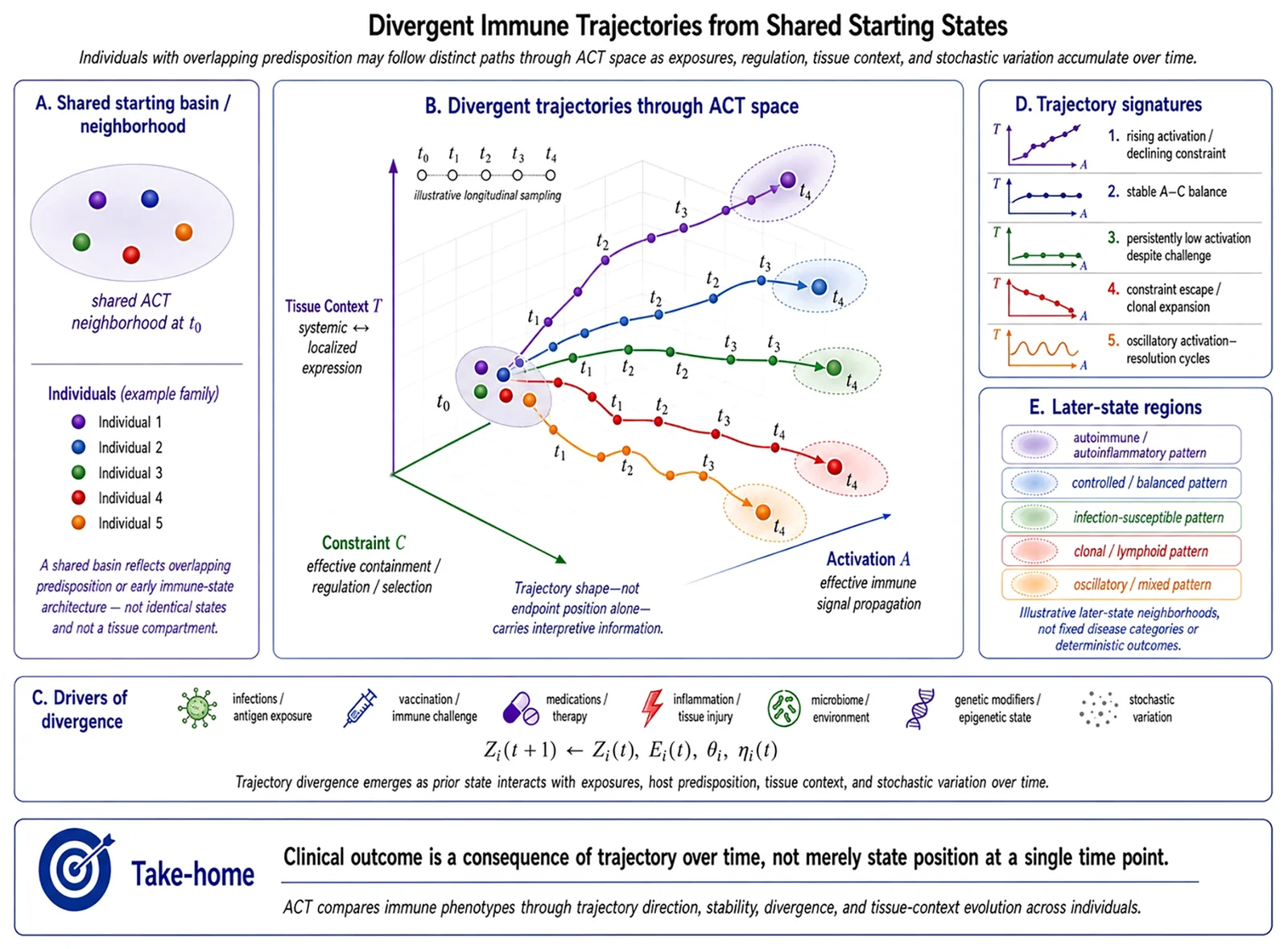
Divergent immune trajectories from shared starting states. **(A)** Five related individuals beginning in a shared ACT neighborhood at t₀. **(B)** Divergent trajectories through ACT space over illustrative longitudinal sampling points t₀–t₄. **(C)** Drivers of divergence, including exposures, therapy, tissue injury, microbiome, genetic modifiers, and stochastic variation. **(D)** Representative trajectory signatures. **(E)** Illustrative later-state regions, which are not fixed disease categories or deterministic outcomes.

### 2.5 Clinical interpretation and connection to practice

ACT formalizes reasoning clinicians already practice: integrating labs, course, family history, and longitudinal change into an implicit model of immune behavior over time. Observed data map to clinical and molecular measurements; estimated state to the clinician’s inferred model; trajectories to longitudinal patterns of flare, remission, or progression; cross-individual comparison to identifying shared and divergent immune patterns. Clinicians already reason in terms like “high activation” or “clonal escape” without numerical precision; ACT preserves this while embedding it in a structure compatible with computational modeling.

### 2.6 Summary of the mapping

Together these components define a structured progression: data → state → trajectory → pathway prioritization. ACT does not claim immune biology is truly three-dimensional, only that Activation, Constraint, and Tissue Context form a clinically interpretable reduced state space through which immune behavior can be tracked, compared, and translated into pathway-level hypotheses. The following section extends this representation longitudinally, treating immune phenotype not as a static coordinate but as a trajectory through reduced immune-state space.

## 3 Trajectory and pathway-level reasoning

### 3.1 Immune phenotype as trajectory

Section 2 established ACT as a reduced coordinate system for representing immune state at a given point in time. Longitudinally, however, immune phenotype is not a single position within ACT space, but a trajectory: a sequence of estimated states $\hat{\text{Z}}$(t) = (Â(t), Ĉ(t), $\hat{\text{T}}$(t)) observed or inferred across successive time points.

A trajectory captures not only where an individual’s immune state lies at a given moment, but how that state changes over time — whether activation is rising or resolving, whether constraint is holding or failing, and whether tissue involvement is expanding, contracting, or shifting between compartments. This is consistent with the probabilistic state-update structure in **Figure 4**, in which each successive estimate depends on the prior state, observed inputs and exposures, and stochastic influences — with uncertainty accumulating and trajectories diverging from similar starting states as this update is repeated over time.

**Figure 4:**
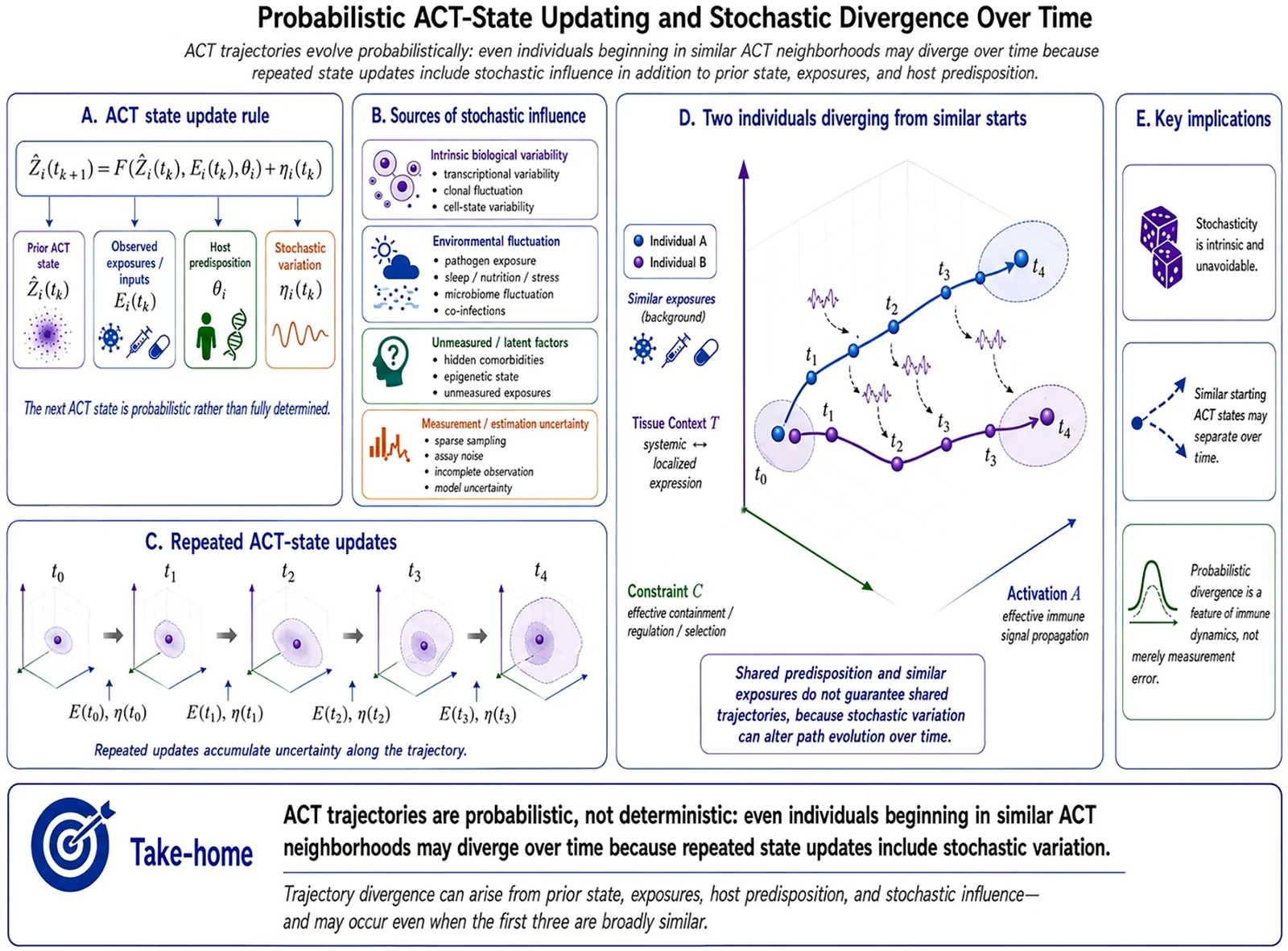
Probabilistic state updating and stochastic divergence in ACT space. **(A)** ACT state update rule, in which the next state depends on the prior state, observed exposures, host predisposition, and stochastic variation. **(B)** Sources of stochastic influence: intrinsic biological variability, environmental fluctuation, unmeasured or latent factors, and measurement or estimation uncertainty. **(C)** Repeated state updates accumulating uncertainty along a trajectory. **(D)** Two individuals with similar starting states and exposures diverging over time. **(E)** Key implications of probabilistic trajectory evolution.

This perspective is consistent with broader developments in systems biology and computational immunology, where biological processes are increasingly represented as movements through state space rather than transitions between fixed categories (19, 20), a perspective ACT applies at the level of clinically interpretable immune phenotype rather than molecular or cellular state.

Framed this way, clinically meaningful events correspond to identifiable patterns of movement through ACT space rather than isolated snapshots. A trajectory in which activation progressively increases while constraint remains stable implies a different underlying process than one in which activation remains unchanged but constraint progressively declines; identical endpoint states may arise from substantially different trajectories. It is therefore the shape, direction, stability, and evolution of movement through reduced state space that provides the primary basis for pathway-level interpretation.

### 3.2 Metastability and trajectory archetypes

Trajectories through ACT space are not unconstrained. In practice, immune behavior frequently settles into recurring patterns of activation, constraint, and tissue localization that persist over time despite ongoing fluctuations in individual measurements. We describe such configurations as metastable: stable under ordinary perturbation but capable of transitioning to another configuration when sufficient cumulative or threshold-level perturbation occurs — a process we formalize later via Burden, a latent time-integrated variable summarizing accumulated perturbation history. This usage differs from the earlier discussion of metastable host parameters (θ) in Section 1, which describes persistent host characteristics; here, metastability describes a property of the trajectory itself — the tendency of an immune phenotype to occupy and remain within a relatively stable region of ACT space.

Related concepts are widely used throughout systems and developmental biology: complex biological systems often occupy attractor-like regions of high-dimensional state space while retaining the capacity to transition between those regions following sufficient perturbation (21), underlying interpretations of cell-fate determination, lineage commitment, and developmental transitions (19, 20).

**Figure 5** illustrates a set of clinically motivated trajectory archetypes occupying distinct regions of ACT space. Each trajectory originates from a homeostatic baseline and moves toward a region of relative stability — an attractor or potential outcome basin — defined by the balance among activation, constraint, and tissue context. These basins do not represent diagnoses or deterministic outcomes; they represent recurring configurations of immune behavior emerging from the interaction of activation dynamics, regulatory control, tissue localization, and environmental influence. A trajectory characterized by sustained activation and diminishing constraint settles differently from one characterized by blunted activation under excessive constraint; a trajectory marked by compartment-specific constraint failure differs from one in which activation gradually drifts upward while remaining partially regulated. The basin itself is therefore not the disease; it is a representation of the system configuration toward which a trajectory tends to evolve — reflecting a familiar feature of clinical practice, in which patients often settle into recognizable patterns of disease behavior that remain relatively stable until shifted by infection, therapy, environmental exposure, aging, or other major perturbations.

**Figure 5:**
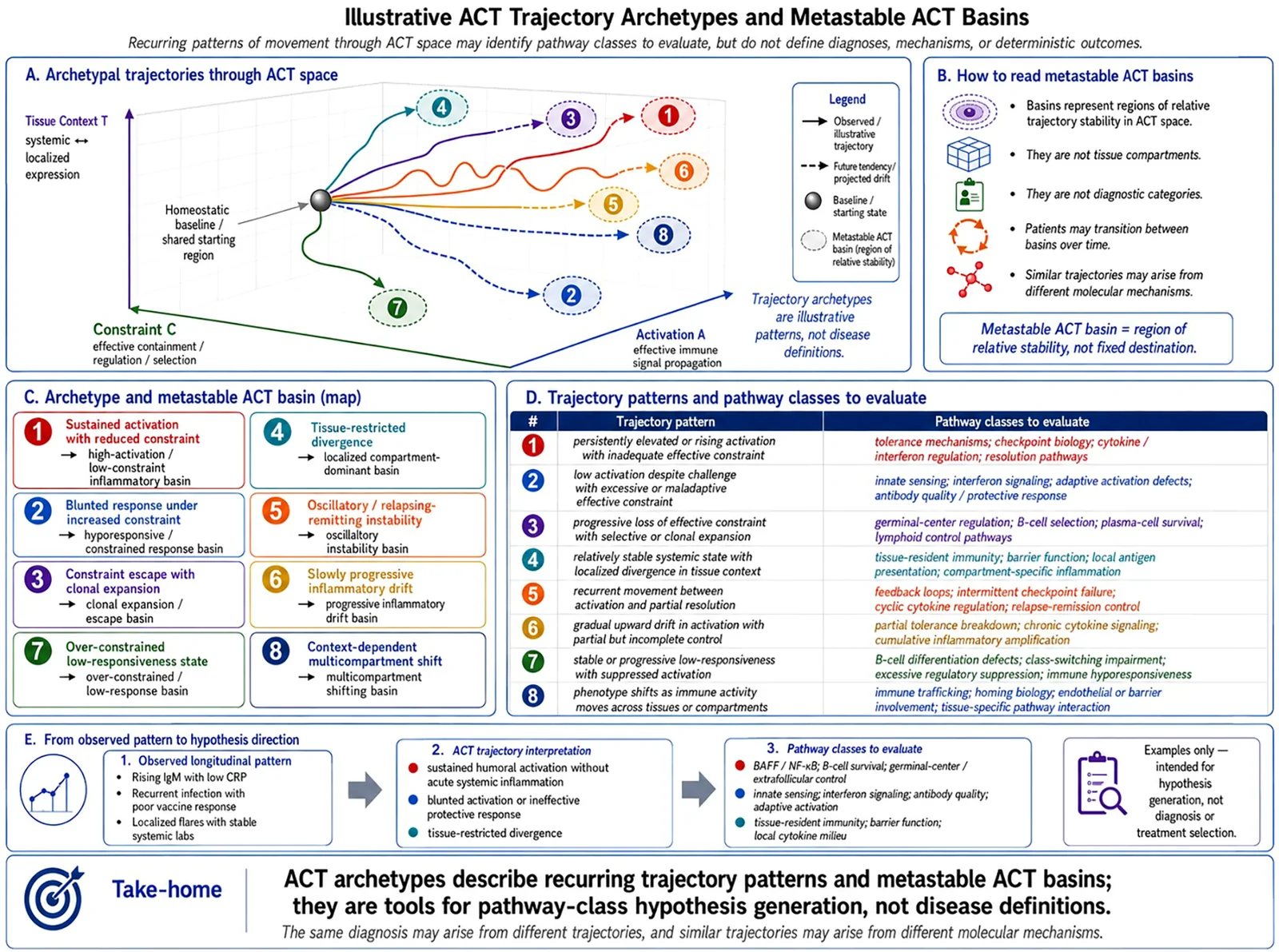
Illustrative ACT trajectory archetypes and metastable outcome basins. **(A)** Archetypal trajectories moving from a shared homeostatic baseline toward metastable ACT basins. **(B)** Guide to reading metastable ACT basins, which are regions of relative trajectory stability rather than tissue compartments or diagnostic categories. **(C)** Map of the eight archetypes and their corresponding basins. **(D)** Trajectory patterns and the pathway classes each suggests for evaluation. **(E)** Example progression from an observed longitudinal pattern to a hypothesis direction, intended for hypothesis generation rather than diagnosis or treatment selection.

### 3.3 Pathway classes and mechanistic interpretation

A central implication of ACT is that pathway classes, rather than individual measurements, may represent an appropriate level of inference from reduced-state trajectory structure. Trajectories may constrain the space of biologically plausible explanations by identifying patterns consistent with dysfunction of specific immune pathways or regulatory programs. ACT alone cannot establish molecular causality, and pathway prioritization remains a hypothesis-generation step rather than a mechanism-identification step. However, when integrated with genetic, molecular, or functional data, ACT-derived trajectory signatures may help prioritize candidate causal lesions within the implicated pathways.

Pathway-level organization has similarly been used to integrate heterogeneous multi-omic findings and relate individual molecular alterations to broader functional programs in precision oncology (22). ACT adopts the same general pathway-class level of description but reaches it differently: rather than identifying molecular drivers from cross-sectional multi-omic data, it uses reduced longitudinal trajectory structure to prioritize pathway classes for independent investigation.

The trajectory archetypes in **Figure 5** illustrate this correspondence. Persistent activation accompanied by reduced constraint may suggest dysfunction within regulatory pathways — tolerance mechanisms, checkpoint signaling, or immune-resolution programs. Blunted activation under increased constraint may instead suggest defects in innate sensing pathways, interferon signaling, or adaptive immune activation. Constraint escape within lymphoid compartments may indicate perturbation of germinal-center control, clonal selection dynamics, or plasma-cell regulation. Tissue-restricted divergence may point toward abnormalities in barrier immunity, tissue-resident immune populations, compartment-specific antigen presentation, or local inflammatory regulation. The remaining archetypes extend these principles to oscillatory, slowly progressive, and multicompartment behaviors; the associated pathway classes are summarized in **Figure 5** and should be read as illustrative rather than diagnostic.

Importantly, this correspondence is functional rather than molecular. Consistent with observations from inborn errors of immunity, similar trajectory structures may arise from distinct molecular perturbations, while similar perturbations may produce divergent trajectories depending on developmental timing, tissue distribution, compensatory pathways, environmental exposure, and stochastic variation (7, 8). This distinction matters in immune-mediated disease broadly, where traditional diagnostic categories often fail to capture underlying biological continuity, and immune phenotypes frequently exist along continua rather than within discrete disease boundaries (23, 24). Rather than asking whether two individuals share a diagnosis, ACT asks whether they occupy related regions of state space and follow related immune trajectories — with the trajectory itself becoming a source of mechanistic information not because it reveals causality directly, but because it constrains which classes of mechanisms remain plausible.

As with the archetypes themselves, these correspondences are illustrative rather than deterministic; real patients may transition between archetypes, occupy intermediate regions, or follow paths not represented by any single pattern. The ACT archetypes are structured tools for interpretation and hypothesis generation, not disease definitions.

An additional limitation is that metabolic state is not explicitly modeled within the current Tri-Axis representation. Immunometabolic programs strongly influence immune-cell activation, differentiation, persistence, and functional specialization; in the present formulation these effects are represented indirectly through their influence on Activation, Constraint, and Tissue Context rather than through an independent state dimension. ACT may therefore incompletely capture situations in which metabolic regulation is itself the dominant driver of immune behavior — a question for future work.

## 4 Illustrative worked example: humoral–neural immune trajectory

A clinically relevant example of translational complexity is the overlap between chronic inflammatory demyelinating polyneuropathy (CIDP), anti-myelin-associated glycoprotein (anti-MAG) neuropathy, monoclonal gammopathy–associated neuropathy, and related immune-mediated neurologic disorders. These conditions may present with similar clinical phenotypes — sensory dysfunction, progressive neuropathy, gait impairment, disability — while their underlying biological drivers differ substantially, spanning inflammatory, autoimmune, humoral, clonal, and lymphoproliferative mechanisms (25). Similar presentations may emerge from distinct pathways, while similar pathway disturbances may produce different clinical outcomes depending on tissue involvement, timing, environmental exposure, and host susceptibility.

For patients and families, this complexity often manifests as a prolonged diagnostic journey across neurology, immunology, hematology, rheumatology, and infectious disease, with each specialty appropriately focused on the manifestation most visible within its domain: neuropathy as a neurologic disorder, recurrent infection as an infectious-disease problem, monoclonal gammopathy as a hematologic finding, inflammatory manifestations as autoimmune disease. Each assessment may be individually correct while the broader longitudinal pattern remains unexplained — patients frequently experience these manifestations not as independent conditions, but as successive expressions of a single unresolved process evolving over time. Traditional diagnostic frameworks, optimized for classification within specific disease domains, do not by themselves address whether neuropathy, recurrent infection, inflammatory flares, monoclonal gammopathy, and autoimmune manifestations reflect related expressions of a common evolving trajectory. ACT is designed as a framework for exploring that possibility — not replacing diagnosis, but organizing observations from multiple specialties into a shared representation of immune behavior over time. Section 4.4 returns to this diagnostic-fragmentation problem directly, after the worked example below has given it concrete shape.

This challenge extends into treatment selection. B-cell depletion with rituximab attempts to modify pathogenic humoral populations directly, whereas broader immunomodulatory approaches such as intravenous immunoglobulin (IVIG) influence immune regulation through multiple mechanisms, including effects on Fc-receptor signaling, cytokine networks, and humoral regulatory pathways (26, 27). Pathways supporting B-cell survival and persistence, including BAFF-mediated signaling, may represent additional candidate intervention points in disorders characterized by sustained humoral activation (28). Within a reduced-state framework, evidence of persistent humoral activation — supported by longitudinal course, family history, and convergent observations across specialties — can be interpreted as a trajectory feature rather than a disease label. The same underlying humoral trajectory may ultimately manifest as autoimmune neuropathy, anti-MAG neuropathy, monoclonal gammopathy-associated neuropathy, lymphoproliferation, MGUS, or plasma-cell malignancy depending on the interaction between activation, constraint, tissue context, and stochastic biological events over time (25, 29). ACT does not attempt to predict which specific outcome will occur. Rather, it provides a framework for understanding how apparently distinct clinical endpoints may emerge from a shared underlying pattern of immune-state behavior. Section 4.5 returns to these same pathway classes as intervention targets, once the trajectory has been reconstructed below.

In ACT terms, such a pattern corresponds to a state characterized by elevated activation within humoral pathways, insufficient effective constraint on B-cell persistence, and a systemic humoral driver with tissue-restricted clinical manifestations — a trajectory that narrows the space of plausible mechanistic explanations and intervention strategies without determining either.

Note: This example is illustrative rather than prescriptive; clinical decisions remain the responsibility of the treating physician and require independent diagnostic, molecular, functional, and therapeutic evaluation.

### 4.1 Trajectory reconstruction

Similar patterns of incomplete penetrance, variable expressivity, and divergent immune-mediated outcomes have been described across inborn errors of immunity (IEI) and related immune dysregulation syndromes, in which infectious susceptibility, autoimmunity, inflammatory disease, and lymphoproliferative phenotypes may emerge from shared pathway-level vulnerabilities while differing substantially in clinical presentation (6, 8, 30).

Consider a specific instance of this pattern: an older individual with lifelong recurrent mucosal infections, later vaccine-associated inflammatory flares, progressive anti-MAG neuropathy, and increasing IgM MGUS without overt hematologic malignancy. Conventional reasoning separates these into infection history, inflammatory episodes, peripheral neuropathy, and hematologic surveillance. ACT instead asks whether they represent sequential phases of a shared immune-state trajectory characterized by chronic humoral activation, incomplete or maladaptive constraint, and evolving tissue-context expression:

Early baseline — Observations: lifelong recurrent mucosal infections. Activation: recurrent antigenic stimulation; chronic barrier engagement. Constraint: incomplete resolution or impaired containment. Tissue basin: mucosal/barrier. Interpretation: early evidence of persistent activation preceding formal diagnosis.

Immune perturbation — Observations: vaccine-associated inflammatory flares. Activation: increased activation following antigenic challenge. Constraint: delayed or incomplete return to baseline. Tissue basin: systemic inflammatory. Interpretation: suggests trajectory instability rather than isolated vaccine response.

Humoral–neural phenotype — Observations: progressive neuropathy, anti-MAG antibodies, rising IgM MGUS, no overt malignancy. Activation: persistent B-cell and humoral activation. Constraint: altered tolerance, autoreactive persistence, or clonal escape. Tissue basin: peripheral nerve plus marrow/B-cell. Interpretation: tissue-specific expression of unresolved activation–constraint imbalance.

Familial divergence — Observations: relatives expressing distinct immune-mediated endpoints. Activation: shared activation tendency may be present. Constraint: constraint failure varies across individuals, exposures, and modifiers. Tissue basin: different dominant basins. Interpretation: variable expressivity arising from related trajectory architecture. (Section 4.3 develops this row in full.)

Constraint here is inferred narratively from clinical course rather than direct measurement; prospective application of ACT would require independent measures — regulatory T-cell frequency, checkpoint-marker expression, exhaustion signatures, tolerance assays — rather than retrospective inference.

### 4.2 Falsifiability of the trajectory interpretation

The value of a trajectory-level interpretation depends on its capacity to be wrong. Under ACT, persistence of tissue-level consequence requires persistence of unresolved activation–constraint imbalance rather than activation alone: had constraint remained adequate at each phase — for example, if vaccine-associated flares reliably resolved without residual autoantibody generation, clonal expansion, or lasting tissue consequence — the framework would predict no cumulative trajectory should emerge, regardless of how often antigenic stimulation occurred. Likewise, recurrent mucosal infections and repeated vaccine responses occurring without subsequent humoral, neurologic, autoimmune, or clonal manifestations would represent a contained trajectory rather than a suppressed one, and would argue against the trajectory interpretation for that individual. The purpose of ACT is not to explain all immune phenotypes, but to generate testable predictions about when longitudinal immune patterns should and should not emerge.

### 4.3 Familial divergence and variable expressivity

Returning to the familial-divergence row introduced in 4.1: the explanatory value of ACT depends on divergence being demonstrable rather than assumed. Consider a sibling who shares the same early pattern of recurrent mucosal infection but maintains adequate constraint following antigenic challenge: inflammatory events resolve completely, no persistent autoantibody signal develops, and no clonal expansion emerges, leaving a trajectory that remains predominantly infection-focused. A second relative might begin from a similar activation profile yet experience a different pattern of regulatory failure, ultimately expressing lymphoproliferative or plasma-cell–dominant disease rather than neural involvement.

Under conventional frameworks these individuals may appear to have unrelated disorders. ACT instead asks whether they represent divergent trajectories emerging from a partially shared immune-state configuration — consistent with the growing recognition that IEI-associated phenotypes often display substantial variable expressivity and incomplete penetrance (7, 8). The question shifts from “Do these individuals have the same diagnosis?” to “Do they occupy related regions of immune-state space and diverge because activation, constraint, and tissue-context interactions evolve differently over time?”

### 4.4 Clinical interpretation and the translational gap

The diagnostic fragmentation described above is not a failure of any individual specialty — each domain-specific assessment in the case above (infectious, hematologic, neurologic, autoimmune) is independently defensible. ACT does not replace conventional evaluation for hematologic malignancy, neuropathy subtype, infectious susceptibility, autoimmune mimics, or alternative explanations for tissue injury, and does not automatically answer whether a given patient’s observations are related. What it offers instead is a structure in which apparently disconnected observations can be evaluated within a common representational framework — organizing immune behavior as a trajectory through Activation, Constraint, and Tissue Context space, and creating a bridge between specialty-specific observations and longitudinal, system-level interpretation. Recurrent infection, inflammatory flares, rising monoclonal signal, autoantibody production, and tissue-specific pathology are not assumed to share a mechanism; they become observations positioned along a shared trajectory whose structure may generate testable pathway-level hypotheses — consistent with broader efforts in precision immunology to understand immune-mediated disease as continuum-like dysregulation rather than isolated diagnostic categories (23, 24).

### 4.5 From trajectory to intervention hypotheses

This reframing changes the therapeutic question: instead of asking only how to suppress the current manifestation, the framework asks whether the underlying trajectory itself can be redirected before additional downstream manifestations emerge. In the present example, persistent humoral activation together with progressive IgM MGUS, anti-MAG antibody production, and neural involvement motivates investigation of the upstream survival and activation pathways introduced earlier — BAFF-axis signaling, Fc-receptor and cytokine-network effects, and B-cell persistence mechanisms — as candidates for redirecting the trajectory rather than only suppressing its current manifestation. These pathway classes are prioritized not because they define a diagnosis, but because they represent biologically plausible mechanisms capable of generating the observed trajectory structure through different combinations of Activation, Constraint, and Tissue Context. Consistent with observations from immune dysregulation syndromes, distinct molecular perturbations may converge on similar trajectory architectures, while similar perturbations may produce divergent outcomes depending on timing, tissue involvement, compensatory pathways, and stochastic variation (7, 8, 24). The framework functions as a hypothesis-generation tool rather than a mechanistic inference engine.

### 4.6 Summary

This example illustrates the intended clinical role of ACT: not to determine diagnosis, identify causal mechanisms, or prescribe intervention, but to help identify situations in which years of observations may represent a coherent longitudinal pattern rather than a collection of isolated events. The resulting trajectory can be used to prioritize pathway classes for independent evaluation using genomic, molecular, immunologic, or functional data not used in constructing the trajectory itself — suggesting where to look next without determining what will be found. Most importantly, ACT attempts to provide a common translational language between the way medicine often encounters immune-mediated disease, through specialty-specific manifestations, and the way patients and families experience it: as a single evolving story whose chapters happen to be written in different organs, tissues, and clinical domains.

## 5 Family-level application: shared predisposition and divergent trajectories

### 5.1 From a single case to a general implication

The worked example in Section 4 illustrated familial divergence through one hypothetical family: an index case with a humoral–neural trajectory, alongside relatives whose shared early activation profile evolved toward different outcomes depending on how, where, and to what extent regulatory control was maintained or lost. This pattern is not unique to that example — it reflects a broader principle that immune phenotype is often best understood not only at the level of the individual, but also in the context of shared predisposition and divergent trajectories across related individuals.

Family history is conventionally collected as a catalog of diagnoses, documented independently and interpreted according to established disease categories. This remains clinically useful but may obscure biological relationships when different manifestations arise from a shared predisposition affecting immune activation, regulation, or tissue-specific expression. ACT proposes an alternative: family history as a structured set of related immune behaviors distributed across individuals, where the most informative feature of a family may not be which diagnoses occur, but which immune-state patterns recur and how they diverge over time — reframing family history from a catalog of diseases into a source of information about the structure of immune-state space itself.

### 5.2 Extending the family: a relative who never reaches a clonal outcome

Section 4.3 already showed two branches of this same family diverging from shared early activation: one relative in whom inflammatory events resolve completely and the trajectory remains infection-focused, and another in whom regulatory failure progresses toward lymphoproliferative or plasma-cell–dominant disease. A third relative extends this picture rather than repeating it. This individual experiences recurrent severe infection despite measurable antibody production — no MGUS, no lymphoproliferation, no clonal expansion — yet shows the same persistent humoral activation seen in the other two. Activation remains elevated not because the trajectory has entered a clonal process, but because the response generated is not effectively protective.

Viewed independently, these three relatives suggest three unrelated diagnoses. Viewed through ACT, all three share the same starting signature: sustained, unresolved humoral activation. The shared feature is not diagnosis, severity, or outcome, but the persistence of a common immune-state pattern — family resemblance appears earliest at the level of Activation, Constraint, and Tissue Context, not at the level of disease labels, with trajectories diverging only after that shared state interacts with differences in regulation, environmental exposure, stochastic variation, age, and tissue-specific biology. This reflects a broader class of observations increasingly recognized in studies of immune variability and inborn errors of immunity, where shared genetic predisposition may produce markedly different phenotypic outcomes across affected relatives (6–8).

### 5.3 Reframing the role of family history

These examples suggest a shift in what family history is used for — from “Which diagnoses occur in this family? Which diseases cluster together?” to “Do these individuals share a common pattern of immune behavior? How do their trajectories diverge across infection, autoimmunity, inflammation, clonal processes, or tissue-specific disease? Which pathway classes are compatible with both the shared starting state and the observed divergence?” The central question becomes not “What disease does this family have?” but “What trajectory architecture does this family share?”

This is the family-level analogue of the individual-level argument developed earlier: just as a single trajectory narrows the space of plausible pathway classes for one patient, a collection of divergent trajectories across a family can narrow that space further by revealing which pathway disturbances are compatible with a shared immune-state foundation and which are not. The third relative above is the clearest illustration of why this matters clinically: under conventional evaluation this individual may appear least concerning, since no clonal marker, malignancy, or autoimmune pathology is present. ACT instead treats the shared activation pattern itself as biologically important — the absence of a clonal endpoint does not erase the shared starting state, and recognizing that shared state keeps the family’s underlying vulnerability visible even in the relative whose presentation appears comparatively benign.

### 5.4 Implications for clinical and research practice

Practically, this suggests that family history collection might productively move beyond “What diagnoses run in this family?” toward “What pattern of activation, constraint, and tissue-context divergence do these diagnoses collectively suggest?” — a question standard diagnostic intake is rarely structured to answer. In the example above, recognizing a relative’s neuropathy and another relative’s plasma-cell malignancy as related expressions of a shared activation pattern could plausibly motivate different surveillance or earlier, broader pathway-focused evaluation in the third family member whose presentation currently appears limited to recurrent infection — precisely because that presentation, reframed through a shared trajectory rather than read in isolation, is not actually the mildest case in the family so much as the least progressed one.

### 5.5 Summary

The family-level application of ACT extends the framework into the space where many immune-mediated diseases are first recognized: patterns observed across related individuals. It does not claim that family members share a diagnosis, nor that shared phenotypes imply a shared mechanism — only that related individuals may occupy neighboring regions of immune-state space while following distinct trajectories shaped by regulation, environment, timing, stochastic variation, and tissue-specific biology. Divergence is not an obstacle to interpretation; it is part of the signal. The contribution of family history becomes not merely the identification of recurring diagnoses, but the reconstruction of shared trajectory architecture — extending translational reasoning from the individual to the family, and treating family history not as background information but as an additional source of evidence about how immune-state trajectories emerge, diverge, and become visible across clinical domains.

## 6 Toward computational operationalization

### 6.1 Purpose and scope

The preceding sections presented ACT as a translational coordinate system for reasoning about immune-state trajectories. This section addresses how the reduction map φ and the resulting trajectory structure might be operationalized computationally, even in advance of full empirical validation. What follows is a framework-level specification, not a fitted or validated predictive model: its purpose is to demonstrate that ACT’s central constructs — activation, constraint, tissue context, and their evolution over time — can be expressed in a bounded, internally consistent, falsifiable computational form, rather than remaining solely qualitative. The scaffold was developed through detailed examination of a shared B-cell survival signaling node whose disruption maps onto multiple clinically distinct downstream conditions; the ACT reduction was derived by observing recurring structure in that examination, not fitted to patient or population data.

This section is best understood as an initial and deliberately incomplete specification rather than a finished computational model. Its aim is to show that ACT’s constructs can be given an explicit, bounded, and internally consistent dynamical form — not to settle the form each component should ultimately take. Several choices made here are provisional and are noted as such: some quantities are represented as inputs rather than through their own governing equations, certain functions are specified only up to their qualitative shape, and various extensions — richer estimation, inter-compartment coupling, endogenous dynamics for additional components — are identified but not developed. These are left open for future work to debate, refine, and decide. What the section establishes is that the framework admits a coherent computational expression; how best to elaborate that expression is a separate and ongoing question.

### 6.2 Estimating latent state and reducing to ACT coordinates

We distinguish two steps previously compressed into a single mapping. First, ψ estimates the latent immune state from heterogeneous clinical, laboratory, and historical observations: ψ(x_i_(t)) → S_i_(t). The clinically reportable ACT coordinates are then obtained by the reduction map φ, applied to this estimated latent state:


$${\text{Z}}_{\text{i}}\left(\text{t}\right)=\text{φ}\left({\text{S}}_{\text{i}}\left(\text{t}\right)\right)=\left({\text{A}}_{\text{i}}\left(\text{t}\right),{\text{ C}}_{\text{i}}\left(\text{t}\right),{\text{ T}}_{\text{i}}\left(\text{t}\right)\right)$$

where A_i_(t), C_i_(t), and T_i_(t) represent Activation, Constraint, and Tissue Context for individual i at time t. This distinction matters because the full latent state S_i_(t) may contain additional internal quantities, estimated by ψ and shaping trajectory evolution, that do not themselves become additional ACT axes under φ.

Activation reflects the degree of immune drive present in the system — humoral activation, inflammatory activity, recurrent antigen-driven stimulation, effector signaling. Constraint reflects effective containment — regulatory, tolerance, checkpoint, selection, and resolution mechanisms. Pathological narrowing of the immune repertoire — monoclonality, clonal dominance, repertoire collapse — does not represent high Constraint; it is a possible downstream consequence of Constraint having been inadequate relative to sustained Activation over time. Constraint, as defined here, is containment, not restriction, and does not distinguish between deficient regulatory quantity and deficient regulatory quality: a germinal-center-deficient or extrafollicular-dominant humoral response may present as reduced effective Constraint despite normal or elevated regulatory cell numbers, since containment is defined by functional outcome rather than by which cellular pathway produced it. Distinct mechanisms — insufficient regulatory-cell number, adequate number with impaired selection quality, or premature resolution of an otherwise appropriate response — may therefore project onto similar regions of reduced Constraint, consistent with the general principle, developed further in Section 7.3, that ACT coordinates compress mechanistically distinct processes into common effective quantities.

Tissue Context represents the systemic or compartment-localized field within which activation and constraint are expressed, consistent with its definition in Section 2: immune-state behavior is expressed through tissues with distinct exposure histories, resident immune populations, barrier functions, stromal environments, repair states, and vulnerabilities.

Neither ψ nor φ diagnoses. Together they organize heterogeneous observations — immunoglobulin patterns, cell counts, infection history, autoimmune history, vaccine or titer behavior, tissue-specific findings — into interpretable quantities. Consistent with the standardization principle introduced in Section 2.3, raw observations are first converted into directionally meaningful abnormality signals before being combined, preventing incompatible measurement types from being averaged as though equivalent.

For notational simplicity, the individual index i is suppressed in the compartment-level equations below; A_k_(t), C_k_(t), T_k_(t), and B_k_(t) should be read as A_ik_(t), C_ik_(t), T_ik_(t), and B_ik_(t) for individual i and compartment k.

### 6.3 Net pressure and accumulated burden

Within compartment k, we define net immune pressure as I_k_(t) = max(0, A_k_(t) − C_k_(t)) — zero whenever effective constraint matches or exceeds activation, and positive only when activation exceeds available containment. Sustained or repeated net pressure does not immediately constitute tissue pathology; rather, it may accumulate as a latent, time-integrated quantity we term Burden, B_k_(t):


$${\text{B}}_{\text{k}}\left(\text{t}+1\right)={\text{B}}_{\text{k}}\left(\text{t}\right)+\text{α}\cdot {\text{I}}_{\text{k}}\left(\text{t}\right)\cdot \left(1-{\text{B}}_{\text{k}}\left(\text{t}\right)\right)-\text{ρ}\cdot {\text{C}}_{\text{k}}\left(\text{t}\right)\cdot {\text{B}}_{\text{k}}\left(\text{t}\right)+{\text{η}}_{\text{k}}\left(\text{t}\right)$$

The term α · I_k_(t) · (1 − B_k_(t)) allows Burden to accumulate in proportion to unresolved pressure while self-limiting as B_k_(t) approaches its upper bound, reducing reliance on *post hoc* clipping. The term ρ · C_k_(t) · B_k_(t) represents decay or resolution proportional to currently available constraint, allowing Burden to recede when containment is restored; η_k_(t) represents stochastic variation.

Burden is not proposed as a fourth ACT axis. The full immune state S_t_ is understood to be higher-dimensional than its reduced projection Z_t_ = φ(S_t_) = (A, C, T); Burden is one latent quantity, estimated as part of S_t_, that shapes trajectory evolution without itself being reported as an ACT coordinate. This mirrors the resolution adopted for Tissue Context in Section 2.1: a single interpretable coordinate at clinical resolution may resolve into compartment- and history-dependent structure at computational resolution.

### 6.4 Tissue evolution as a function of burden

Tissue Context is not static once immune pressure has been expressed within a compartment. We represent its evolution as a bounded function of accumulated Burden:


$${\text{T}}_{\text{k}}\left(\text{t}+1\right)={\text{T}}_{\text{k}}\left(\text{t}\right)+\text{γ}\cdot \text{g}\left({\text{B}}_{\text{k}}\left(\text{t}\right)\right)\cdot \left(1-{\text{T}}_{\text{k}}\left(\text{t}\right)\right)+{\text{η}}_{\text{k}}\left(\text{t}\right)$$

where g(·) describes how sustained accumulated imbalance shifts a compartment’s susceptibility over time — through remodeling, altered antigen exposure, loss of local regulatory reserve, stromal change, vascular alteration, or persistent local immune memory. The factor (1 − T_k_(t)) prevents unbounded escalation. This formalizes the claim from Section 2.1: tissue context is not merely a site of expression, but a record of prior activation-constraint history that conditions future vulnerability within that compartment.

In the present formulation, compartments evolve independently; explicit inter-compartment coupling is the immediate next extension.

### 6.5 Sensitized activation: a feedback loop

Accumulated Burden may also alter the sensitivity of future activation to renewed exposure, represented as a multiplicative gain on external exposure rather than as autonomous, trigger-independent activation:


$${\text{A}}_{\text{k}}\left(\text{t}+1\right)={\text{A}}_{\text{k}}\left(\text{t}\right)+\text{β}\cdot {\text{E}}_{\text{k}}\left(\text{t}\right)\cdot \left(1+\text{σ}\cdot {\text{B}}_{\text{k}}\left(\text{t}\right)\right)\cdot \left(1-{\text{A}}_{\text{k}}\left(\text{t}\right)\right)-{\text{β}}_{\text{u}}\cdot {\text{U}}_{\text{k}}\left(\text{t}\right)+{\text{η}}_{\text{k}}\left(\text{t}\right)$$

Here, E_k_(t) represents an external exposure, and the term (1 + σ · B_k_(t)) captures the possibility that a compartment carrying prior unresolved burden responds disproportionately to a subsequent exposure of otherwise ordinary magnitude — the same trigger producing a larger consequence than in an unburdened compartment, with the response itself becoming the source of tissue injury. We restrict this formulation to an amplifying rather than autonomous mechanism deliberately: we do not claim Burden alone generates activation absent exposure, only that accumulated Burden lowers the threshold or increases the gain by which ordinary exposure produces disproportionate response — sensitization rather than a breakdown of homeostasis.

The subtracted term β_u_ · U_k_(t) represents an active intervention — regulatory, therapeutic, or otherwise externally applied — capable of reducing activation directly, with β_u_ governing its effectiveness. We define this term as an intervention input rather than an endogenous resolution process deliberately: the present formulation contains no separate mechanism by which activation decays on its own once an eliciting exposure has ended. This is an intentional simplification of the scaffold, not a claim that immune activation never resolves spontaneously. In post-infectious immune-mediated disease, the inciting exposure may no longer be detectable while immune-mediated injury persists; Guillain–Barré syndrome provides a clinically familiar example in which immune activation can continue after an antecedent infection and may require immune-directed treatment such as intravenous immunoglobulin or plasma exchange in clinically significant cases (31). Under the present scaffold, Activation is therefore sticky by default: absent U_k_(t), a compartment that has crossed into sustained activation may remain there, and Burden may continue to accumulate for as long as I_k_(t) remains positive. Whether particular biological contexts additionally warrant an endogenous decay term — activation waning gradually without intervention — is a question the current scaffold leaves open rather than answers; Section 6.10 tests only the intervention-dependent form specified here.

### 6.6 A causality constraint

The dynamic system above is coherent only if a specific ordering is respected: quantities computed at time t may influence quantities at time t+1, but must never influence other quantities within the same time step. Same-time mutual inflation — activation raising Burden, which then raises activation within a single step — is circular and produces no genuine explanatory content; cross-time feedback, in which prior accumulated state shapes subsequent response, is the ordinary structure of a history-dependent biological system. This is enforced throughout:


$${\text{A}}_{\text{k}}\left(\text{t}\right),{\text{ C}}_{\text{k}}\left(\text{t}\right)\to {\text{I}}_{\text{k}}\left(\text{t}\right)\to {\text{B}}_{\text{k}}\left(\text{t}+1\right)$$

$${\text{B}}_{\text{k}}\left(\text{t}\right)\to {\text{T}}_{\text{k}}\left(\text{t}+1\right)$$

$${\text{B}}_{\text{k}}\left(\text{t}\right),{\text{ E}}_{\text{k}}\left(\text{t}\right),{\text{ U}}_{\text{k}}\left(\text{t}\right)\to {\text{A}}_{\text{k}}\left(\text{t}+1\right)$$

No variable at time t is permitted to depend on another variable computed from the same time step in a way that inflates its own value.

### 6.7 Toward family-level comparison

Restoring the individual index suppressed above: where longitudinal ACT trajectories are available for related individuals, comparison across family members should proceed only after each individual’s trajectory has been independently estimated, via ψ, from that individual’s own observations — never the reverse. Representing uncertainty through resampling or Monte Carlo methods, and evaluating shared versus divergent family-level architecture through trajectory shape rather than endpoint diagnosis alone, are natural extensions of this framework, which we flag but do not implement here. We emphasize the discipline such an extension would require: family-level similarity may inform interpretation of independently estimated trajectories, but must never be permitted to feed back into, and thereby inflate, the individual coordinates from which it was derived.

### 6.8 Host predisposition as parameter modulation

The scaffold treats the parameters governing net pressure, burden accumulation, tissue evolution, and sensitized activation — α, ρ, σ, β, β_u_, γ — as fixed for a given individual across a single trajectory. These parameters are distinct from the time-varying inputs E_k_(t) and U_k_(t): exposure and intervention describe what happens to an individual over time, while α, ρ, σ, β, β_u_, and γ describe how that individual’s system responds to what happens — a distinction that matters because two individuals with identical exposure and intervention histories may still diverge if their underlying parameters differ.

Parameters are held fixed within a single trajectory. In this scaffold, an individual’s baseline vulnerability enters through these parameter values — how their system responds to pressure, burden, and exposure — rather than through drift in the parameters over time. Age- or load-structured vulnerability is therefore represented as the interaction of fixed individual parameters with accumulating burden and exposure, not as time-dependent change in the parameters themselves. Genuine age-related change in the parameters — as in immunosenescence — is not modeled here and represents a natural extension.

Host genetic predisposition, including monogenic lesions of the kind characterized in the human genetics of infectious disease, is one source of these underlying parameters rather than a competing account of disease. This is consistent with the observation that monogenic infections may be incompletely penetrant, variably expressive, dependent on microbial exposure, modified by genetic background, and sometimes mirrored by autoimmune mechanisms targeting the same host-defense component (6–8, 32).

This is consistent with the role already assigned to θ in Section 1. A defect affecting a specific regulatory or checkpoint pathway need not manifest as a categorically distinct explanation for disease; it may instead correspond to a structurally altered value of ρ — a reduced capacity to resolve accumulated burden once activation has exceeded constraint — or an altered σ, shifting how readily prior burden sensitizes subsequent activation. For example, TRAF3-mediated restraint of BAFF/APRIL-driven noncanonical NF-κB signaling functions as a checkpoint on B-cell survival signaling; impairment at this node could plausibly correspond to reduced ρ, without requiring a distinct explanatory model for each downstream tissue-specific manifestation. Two individuals carrying the same monogenic lesion would share the corresponding parameter shift, yet could still diverge in clinical trajectory depending on exposure history, the compartment in which pressure accumulates, and stochastic variation — consistent with the variable expressivity and incomplete penetrance widely documented for such lesions (6–8).

We do not propose that a given monogenic lesion can be mapped to a specific parameter or magnitude of shift from present evidence; this would require independent functional and genomic characterization beyond the scope of this framework. We propose only that the scaffold has a principled location for host genetic predisposition to enter — as modulation of trajectory dynamics, with monogenic predisposition as one route by which the parameters producing a given trajectory come to take the values they do.

### 6.9 An illustrative prediction

Consider an adult who develops a pathogen-specific invasive infection outside the age windows classically associated with either monogenic defects of protective immunity or autoimmune phenocopies (8). This does not challenge that model; it extends the question only to cases that remain unexplained after both mechanisms have been considered. ACT predicts that some such cases may reflect accumulated activation–constraint imbalance superimposed on host defense pathways, rather than requiring a newly identifiable single-gene lesion in every instance. This prediction is falsifiable: affected individuals should show longitudinal evidence of sustained or recurrent immune activation, impaired regulatory constraint, or tissue-specific amplification that precedes and is disproportionate to the observed pathogen-specific outcome. Absent such evidence, this application of the framework would be unsupported for that case.

This example is offered to illustrate the kind of hypothesis ACT is intended to generate, not as an explanation for otherwise-unexplained infection. Consistent with the caution that unexplained infections should remain labeled as unexplained until a specific cause is demonstrated, ACT should not be used to replace genetic, immunologic, molecular, or functional evaluation, which remains the appropriate first step for any otherwise-unexplained pathogen-specific susceptibility.

### 6.10 Illustrative computational demonstration

To assess whether the computational scaffold specified above behaves coherently when executed, we implemented the equations in Sections 6.3–6.5 as an illustrative synthetic simulation. The purpose of this exercise was not empirical validation or prediction, but internal coherence testing: whether the bounded, causally ordered system remains stable, accumulates Burden only under sustained net pressure, and produces qualitatively distinct trajectories under different constraint conditions. This follows established precedent for using abstracted computational demonstration to test the internal coherence of a mechanistic hypothesis rather than to validate a predictive model (33).

The simulation used a single tissue-context compartment and three synthetic configurations: a balanced configuration in which constraint remained sufficient throughout; an activation-predominant configuration in which constraint remained insufficient; and a constraint-restored configuration in which resolution was introduced after an initial period of imbalance. No parameter was fit to patient or population data. Across 300 stochastic realizations per configuration, all state variables remained bounded, and the predicted qualitative ordering was observed: balanced trajectories accumulated the least Burden, activation-predominant trajectories accumulated the most, and constraint-restored trajectories showed partial resolution after restoration of containment. Full parameter values, exposure schedules, random seeds, code, extended Monte Carlo results, and visualization outputs are provided in the **Supplementary Material**.

These simulations establish only that the proposed equations can execute as a bounded and causally ordered computational object. They do not demonstrate empirical validity, clinical accuracy, or predictive performance. Parameter values and synthetic case definitions were chosen for illustration and were not derived from real individuals. The demonstration is therefore best understood as a minimal computational sanity check — a necessary but insufficient step toward the prospective empirical validation described above.

Its role is analogous to the framework’s broader logic: to show that a simplified architecture can be made explicit, bounded, and testable without claiming that the simplified representation is itself the full biology.

## 7 Limitations

### 7.1 The exclusion of metabolic state

The most difficult conceptual decision in developing ACT was whether metabolic state should be represented explicitly as a fourth axis rather than an implicit modifier of Activation, Constraint, and Tissue Context. This decision is not considered fully resolved. Immunometabolic programs are often proximal determinants of immune behavior: glycolytic and oxidative metabolic programs influence effector versus regulatory T-cell differentiation, macrophage polarization, lymphocyte persistence, and exhaustion states, while nutrient availability and local metabolite concentrations shape whether immune responses are sustained, suppressed, or redirected (34–36).

The challenge is that metabolic state does not behave like an independent coordinate; rather than occupying a position parallel to Activation, Constraint, and Tissue Context, metabolism frequently conditions all three simultaneously, functioning less as an additional axis and more as a substrate upon which the other axes depend. Incorporating it explicitly would require either a fourth coordinate whose independence from the existing three remains biologically uncertain, or a more fundamental reformulation treating metabolic state as a conditioning layer on the entire framework — neither of which could be developed to the same rigor as the present formulation without substantially expanding scope. The current framework therefore assumes major metabolic influences are reflected indirectly through their observable effects on the three axes, an assumption likely to fail when metabolic dysregulation itself becomes the primary driver of immune behavior — as in chronic inflammatory states, obesity-associated immune dysfunction, metabolic syndromes, tumor microenvironments, and persistent exhaustion programs (35, 36). Whether metabolic state is adequately represented through the existing axes, or contributes independent explanatory information requiring extension beyond the current three-axis structure, remains an open priority for future work.

### 7.2 Dependence on available observations

Immune state is not directly observable; the latent state S(t) must be estimated from measurements that are themselves incomplete, noisy, and variable in resolution. A trajectory reconstructed from routine clinical measurements may differ substantially from one estimated using high-dimensional assays such as single-cell transcriptomics, spatial omics, or immune-repertoire sequencing — inferred state can only be as informative as the observations used to estimate it. ACT trajectories should therefore always be interpreted relative to the quality, scope, and resolution of the data from which they are derived; improvements in measurement technology may alter state estimates without implying the underlying biological trajectory has changed. This observer dependence must be acknowledged whenever trajectories are compared across studies, institutions, or time periods, particularly as modern immunology increasingly spans scales from routine clinical observation to single-cell and spatially resolved measurement (16, 17).

### 7.3 Simplification of high-dimensional biology

ACT intentionally compresses an extraordinarily complex biological system into three interpretable dimensions — a design choice, not a claim about the true dimensionality of immune behavior. Each axis combines many distinct biological processes that may not be interchangeable under all circumstances; different molecular pathways, cell populations, and regulatory mechanisms may project into similar regions of ACT space despite different biological causes and consequences. Position within ACT space should therefore not be interpreted as equivalent to mechanism. The framework preserves information useful for interpretation and hypothesis generation; it does not replace mechanistic models of immune function.

### 7.4 Dependence on longitudinal data

ACT is fundamentally a longitudinal framework: trajectory, divergence, metastability, pathway prioritization, and family-level comparison all derive their meaning from observations collected across time. In many clinical settings, however, longitudinal data may be sparse, irregularly sampled, or unavailable, and retrospective reconstruction may be influenced by survivorship bias or selective testing. The framework is expected to perform best when repeated measurements allow meaningful reconstruction of immune behavior over time; conditions characterized by singular acute events or limited longitudinal observation may be less amenable to interpretation using the present framework.

### 7.5 Limits of pathway-level inference

ACT does not directly infer molecular causality. The framework operates at the level of pathway classes, using trajectory structure to constrain the set of biologically plausible explanations — a hypothesis-generation process, not a mechanism-identification process. Different biological mechanisms may generate similar trajectory patterns, while similar molecular perturbations may produce divergent trajectories depending on context, timing, tissue distribution, compensatory pathways, and stochastic variation (7, 8). ACT should not be interpreted as providing evidence of causation; any pathway-level hypothesis it generates requires independent validation using molecular, functional, genomic, or experimental approaches.

### 7.6 Scope and intended use

More broadly, ACT is a conceptual and computational scaffold rather than a complete representation of immune biology. By design, it omits explicit cell-state representation, molecular interaction networks, developmental history, epigenetic regulation, and context-specific signaling structure, prioritizing interpretability, longitudinal reasoning, and translational utility over exhaustive biological completeness. This reflects the central tradeoff underlying the framework: interpretability versus biological completeness. The resulting simplicity is a strength when organizing complex observations, but becomes a weakness whenever omitted dimensions carry substantial explanatory information. ACT should therefore be judged not by whether it captures every aspect of immune biology, but by whether the information it preserves proves useful for understanding, comparing, and reasoning about immune trajectories across individuals, families, and disease domains.

## 8 Conclusion

We propose a framework in which immune phenotype is modeled as a trajectory through a reduced-dimensional state space defined by Activation, Constraint, and Tissue Context. By linking observable data to latent immune state, latent state to longitudinal trajectory, and trajectory to pathway-level hypothesis generation, ACT provides a structured approach for organizing clinical, molecular, and computational reasoning within a common translational coordinate system.

ACT is intentionally reductive. It does not attempt to capture the full complexity of immune biology or directly infer molecular mechanism. Rather, it offers a clinically interpretable structure for reasoning about immune behavior over time. Its central contribution is the treatment of trajectory, rather than static state, as the primary unit of interpretation. Within this view, immune-mediated conditions are understood not as isolated snapshots, but as divergent paths emerging from shared baseline configurations. Cross-individual and family-level trajectory patterns may therefore serve as sources of mechanistic insight by narrowing the space of plausible biological explanations and guiding targeted downstream investigation through genomic, functional, and molecular studies.

ACT is best understood as a scaffold for hypothesis generation, and its value should be judged by the falsifiable hypotheses it produces. The framework would be challenged if distinct reduced-state trajectories failed to correspond to reproducible differences in pathway-level findings across individuals or cohorts. More fundamentally, ACT advances a testable proposition: that trajectory, rather than static state, provides a more informative representation of immune phenotype. If reduced-state trajectories offer no advantage over static snapshots in explaining or predicting divergent immune-mediated outcomes, that proposition fails.

In this sense, ACT proposes not only a framework for reasoning about immune behavior, but also a falsifiable hypothesis about its organization: trajectory may be the phenotype.
