# Supplementary material for: Bringing translational structure to complex immune-mediated conditions: the ACT (Activation–Constraint–Tissue Context) framework for divergent phenotypes
Source: Front Immunol. 2026 Oct 23;17:1866922. doi: 10.3389/fimmu.2026.1866922 (PMC13739710; doi:10.3389/fimmu.2026.1866922)
Supplement: Supplementary file 1 [file Supplementaryfile1.docx]

Supplementary Material

# Computational Demonstration

## Purpose

Section 6 specified a bounded, causally ordered dynamical system implementing the ACT reduction and its associated latent quantities. This section asks a narrow question: does that specification, when executed, behave as claimed—remaining bounded, accumulating Burden only under sustained net pressure, and producing the qualitative trajectory differences the framework predicts? This follows established precedent for using abstracted computational demonstration, rather than empirical validation, to test the internal coherence of a mechanistic hypothesis (An, 2015). We do not ask whether the simulation matches any real patient's data, since it was not constructed from patient data and is not offered as a predictive model.

## Model Construction

We implemented the equations in Sections 6.3–6.5 directly, using a single tissue-context compartment and three synthetic configurations differing only in constraint level and exposure schedule, not chosen to represent real individuals: a balanced configuration in which constraint remained sufficient throughout; an activation-predominant configuration in which constraint remained insufficient throughout; and a constraint-restored configuration, identical to the second at onset, in which constraint and a resolution input were introduced partway through. No parameter was fit to data of any kind; values were selected to produce three clearly separated regimes and adjusted where an initial configuration failed to do so. To assess whether results depended on a single stochastic realization, each configuration was run 300 times with independent random seeds. Full parameter values, exposure schedules, and code are provided in the Supplementary Material.

## Results

Across all 900 simulated trajectories, every state variable remained within its specified bound at all times, with no instance of unbounded growth or collapse. The three configurations produced the predicted ordering—balanced < constraint-restored < activation-predominant—across every quantity examined: peak accumulated Burden, peak Tissue Context shift, and peak Activation on a later, matched-magnitude re-exposure. The constraint-restored configuration accumulated substantial Burden during its initial period of imbalance but showed marked resolution following restoration of constraint, distinguishing it from the sustained elevation seen in the activation-predominant configuration. This ordering was not an artifact of any single run: it held in 300 of 300 stochastic realizations for peak Burden and for sensitized Activation, and in 298 of 300 for peak Tissue-context shift. Figure 6 shows mean trajectories through the reduced coordinate space, with the translucent plane marking the boundary at which net pressure becomes positive.

## Interpretation and Limits

These results establish internal consistency, not empirical validity. Parameter values and case definitions were chosen for illustration and were not derived from or fit to any patient or population dataset. No claim is made that these trajectories resemble any real individual’s clinical course or that this simulation constitutes evidence of ACT’s predictive validity. Its purpose is limited to showing that the framework, as specified, executes as a coherent computational object—a necessary but not sufficient condition for the prospective empirical validation and falsifiable predictions outlined in Section 6.9.

A further limitation concerns the treatment of Constraint in this toy model. In this demonstration, Constraint enters only through its containment and resolution roles: it lowers net immune pressure, defined as one-sided $I=\max(0,A-C)$, and resolves accumulated Burden through the decay term $-\rho CB$, which scales with existing Burden. Accumulation and resolution are therefore represented by distinct terms with appropriate functional forms rather than by a single signed-pressure quantity. Consequently, the present scaffold does not reproduce regimes in which Constraint itself limits protective immunity—the over-constrained, hyporesponsive archetypes described conceptually in Figure 5. Such regimes lie outside the coherence-testing purpose of this demonstration and remain an extension for future work.

Additionally, because additive Gaussian perturbations in this toy model are followed by hard clipping to the interval $\left[ 0 , 1 \right]$, repeated simulations may exhibit minor probability accumulation at the boundaries. This implementation was retained for transparency and simplicity in the present qualitative demonstration; empirical applications could instead use bounded noise distributions, reflected or truncated stochastic processes, or transformed-state formulations that preserve bounded support without boundary accumulation.

Code and Data Availability

Code was developed with the assistance of AI language models (Claude, ChatGPT); all outputs were independently validated by the author against the framework's specified behavior.

# S1. Model Parameters and Configuration Details

Table S1 reports the complete parameter set, initial conditions, and time-varying schedules used to generate the three synthetic configurations reported in Section 6.10. All three configurations share identical values of α, ρ, γ, β, βᵤ, and σ. Configurations differ only in initial conditions, constraint schedule, exposure schedule, and, for S3, the presence of an intervention input. No parameter was fitted to patient or population data. Values were selected to produce three clearly separated qualitative regimes consistent with the illustrative purpose stated in Section 6.10. During revision, the S1 initial configuration was adjusted after an initial pass allowed Activation to exceed Constraint, which was inconsistent with the intended balanced regime.

Table S1. Complete parameter values by configuration.

| **Parameter** | **S1: Balanced** | **S2: Activation-predominant** | **S3: Constraint-restored** |
| --- | --- | --- | --- |
| A(0) | 0.20 | 0.25 | 0.25 |
| C(0) | 0.65 | 0.30 | 0.30 |
| T(0) | 0.20 | 0.20 | 0.20 |
| B(0) | 0.00 | 0.00 | 0.00 |
| Constraint schedule | 0.65 (constant) | 0.30 (constant) | 0.30 for t < 30; 0.58 for t ≥ 30 |
| Exposure schedule | 0.20 (t = 10–13); 0.15 (t = 42–44) | 0.45 (t = 10–23); 0.35 (t = 42–45) | 0.45 (t = 10–23); 0.35 (t = 42–45) |
| Intervention U(t) | None | none | 0.35 (t = 30–41) |
| α (Burden accumulation rate) | 0.18 | 0.18 | 0.18 |
| ρ (Burden decay rate) | 0.08 | 0.08 | 0.08 |
| γ (Tissue evolution rate) | 0.05 | 0.05 | 0.05 |
| β (Activation gain from exposure) | 0.18 | 0.18 | 0.18 |
| βᵤ (Activation reduction from intervention) | 0.08 | 0.08 | 0.08 |
| σ (sensitization coefficient) | 1.20 | 1.20 | 1.20 |
| Noise SD (η, all variables) | 0.015 | 0.015 | 0.015 |
| n_steps | 80 | 80 | 80 |
| Monte Carlo runs | 300 | 300 | 300 |
| Random seed range | 1000–1299 | 1000–1299 | 1000–1299 |

# S2. Full Equation Set

The following equations, introduced in Sections 6.3–6.5 of the main text, were implemented exactly as specified. The individual index i is suppressed throughout, consistent with the notational convention introduced in Section 6.2; all variables below should be read as compartment- and individual-specific (e.g., A(t) ≡ Aᵢₖ(t)).

## Net immune pressure

I(t) = max(0, A(t) − C(t))

## Accumulated Burden (latent; not a reported ACT axis)

B(t+1) = B(t) + α·I(t)·(1 − B(t)) − ρ·C(t)·B(t) + η_B(t)

## Tissue Context evolution

T(t+1) = T(t) + γ·B(t)·(1 − T(t)) + η_T(t)

## Sensitized Activation

A(t+1) = A(t) + β·E(t)·(1 + σ·B(t))·(1 − A(t)) − βᵤ·U(t) + η_A(t)

## Causal ordering constraint

All updates follow strict cross-time dependency: quantities at time t may determine quantities at time t+1, but no quantity is permitted to depend on another quantity computed within the same time step. The enforced dependency structure is:

- A(t), C(t) → I(t) → B(t+1)
- B(t) → T(t+1)
- B(t), E(t), U(t) → A(t+1)

Constraint C(t) is treated as an externally determined schedule in this illustrative demonstration (Table S1) rather than as a variable with its own update equation, consistent with the treatment of host predisposition and intervention as inputs to, rather than outputs of, the scaffold (Section 6.8).

# S3. Extended Monte Carlo Methodology and Results

Each of the three configurations described in Table S1 was simulated 300 times. Each run used an independent random seed (1000–1299) governing the Gaussian perturbation terms η_A, η_B, and η_T (σ_noise = 0.015 for all three). This design was adopted specifically to test whether the qualitative ordering reported in Section 6.10 depended on a single stochastic realization, following the reviewer request to demonstrate how the specified architecture handles stochastic noise over time.

Across all 900 simulated trajectories (300 runs × 3 configurations), all state variables (A, C, T, B) remained finite and within [0,1] at every time step, with no instance of divergence, collapse, or non-finite values.

Table S2. Summary statistics across 300 Monte Carlo runs per configuration.

| **Configuration** | **Peak Burden (mean ± SD)** | **Peak Tissue Context (mean ± SD)** | **Peak Activation, 2nd exposure (mean ± SD)** |
| --- | --- | --- | --- |
| S1: Balanced | 0.090 ± 0.024 | 0.346 ± 0.099 | 0.388 ± 0.094 |
| S2: Activation-predominant | 0.856 ± 0.027 | 0.927 ± 0.049 | 0.910 ± 0.052 |
| S3: Constraint-restored | 0.644 ± 0.045 | 0.771 ± 0.091 | 0.669 ± 0.063 |

Table S3. Robustness of the predicted ordering (S1 < S3 < S2) across independent stochastic realizations.

| **Metric** | **Runs preserving ordering S1 < S3 < S2** | **Percentage** |
| --- | --- | --- |
| Peak Burden | 300 / 300 | 100.0% |
| Peak Tissue Context shift | 298 / 300 | 99.3% |
| Peak Activation on 2nd exposure | 300 / 300 | 100.0% |

The ordering held in every run for peak Burden and peak sensitized Activation, and in 298 of 300 runs for peak Tissue Context shift. The two exceptions reflect ordinary stochastic variation in a bounded system with additive Gaussian noise and do not affect the qualitative interpretation reported in the main text.

# S4. Simulation Code

The complete executable simulation code is reproduced below and is also suitable for direct execution as a separate Python file. The code was drafted and revised with assistance from AI language models (Claude and ChatGPT); all logic, parameter choices, and outputs were independently reviewed and validated by the author against the equations and expected behavior specified in Sections 6 and S2 of this document.

```
"""
ACT toy model -- Section 6.10 computational demonstration
======================================================

Purpose
-------
Instantiate the ACT scaffold specified in Section 6 and verify, via Monte Carlo
simulation across independent noise realizations, that the specified system:

1. remains bounded to [0, 1] for all state variables at all time steps;
2. accumulates latent Burden only when Activation exceeds Constraint;
3. produces the qualitative ordering predicted across three synthetic
   configurations: S1 balanced < S3 constraint-restored < S2 activation-
   predominant; and
4. does so robustly across independent stochastic realizations rather than as
   an artifact of a single random seed.

This script does not fit any parameter to patient or population data. It is not
a predictive or diagnostic model. All parameter values, exposure schedules, and
case definitions were chosen for illustration only.

Reproducibility
---------------
Each Monte Carlo run uses an explicit seed: 1000 + run_index.
"""

import numpy as np
import matplotlib.pyplot as plt


# -----------------------------------------------------------------------------
# Helper functions
# -----------------------------------------------------------------------------

def clip01(x):
    """Clip scalar or array values to the closed interval [0, 1]."""
    return np.clip(x, 0.0, 1.0)


def simulate_case(
    seed,
    n_steps=80,
    A0=0.20,
    C0=0.45,
    T0=0.20,
    B0=0.00,
    alpha=0.18,        # Burden accumulation rate
    rho=0.08,          # Burden decay/resolution rate, proportional to Constraint
    gamma=0.05,        # Tissue Context evolution rate as a function of Burden
    beta=0.18,         # Activation gain from exposure
    beta_u=0.08,       # Activation reduction from intervention/resolution input
    sigma=1.20,        # Sensitization coefficient: Burden amplification of exposure response
    noise_sd=0.015,    # Gaussian perturbation SD applied to B, T, and A each step
    constraint_shift=None,
    exposure_schedule=None,
    intervention_schedule=None,
):
    """
    Simulate one bounded ACT trajectory for a single tissue-context compartment.

    Note:
        This illustrative implementation models one tissue compartment (k = 1),
        with the compartment index suppressed. Full multi-compartment dynamics
        would require parallel compartment-specific state arrays together with
        explicit inter-compartmental spillover and/or trafficking functions.

    Returns arrays for Activation A(t), Constraint C(t), Tissue Context T(t),
    latent Burden B(t), and net immune pressure I(t).

    Causal ordering is enforced explicitly: B(t+1), T(t+1), and A(t+1) are
    each computed only from quantities at time t, never from quantities computed
    within the same step.
    """
    rng = np.random.RandomState(seed)
    exposure_schedule = exposure_schedule or {}
    intervention_schedule = intervention_schedule or {}
    constraint_shift = constraint_shift or {}

    A = np.zeros(n_steps)
    C = np.zeros(n_steps)
    T = np.zeros(n_steps)
    B = np.zeros(n_steps)
    I = np.zeros(n_steps)

    A[0], C[0], T[0], B[0] = A0, C0, T0, B0

    for t in range(n_steps - 1):
        E_t = exposure_schedule.get(t, 0.0)
        U_t = intervention_schedule.get(t, 0.0)

        # Constraint may be externally shifted but is never inflated by
        # same-step Burden or Activation.
        C[t] = clip01(constraint_shift.get(t, C[t]))

        # Net immune pressure.
        I[t] = max(0.0, A[t] - C[t])

        # Latent Burden update.
        B[t + 1] = clip01(
            B[t]
            + alpha * I[t] * (1 - B[t])
            - rho * C[t] * B[t]
            + rng.normal(0, noise_sd)
        )

        # Tissue Context update.
        T[t + 1] = clip01(
            T[t]
            + gamma * B[t] * (1 - T[t])
            + rng.normal(0, noise_sd)
        )

        # Sensitized Activation update.
        A[t + 1] = clip01(
            A[t]
            + beta * E_t * (1 + sigma * B[t]) * (1 - A[t])
            - beta_u * U_t
            + rng.normal(0, noise_sd)
        )

        # Constraint persists unless externally shifted at the next step.
        C[t + 1] = C[t]

    I[-1] = max(0.0, A[-1] - C[-1])
    return A, C, T, B, I


# -----------------------------------------------------------------------------
# Synthetic case definitions
# -----------------------------------------------------------------------------

N_STEPS = 80

EXPOSURE_BALANCED = {
    **{t: 0.20 for t in range(10, 14)},
    **{t: 0.15 for t in range(42, 45)},
}
CONSTRAINT_BALANCED = {t: 0.65 for t in range(N_STEPS)}

EXPOSURE_ACTIVATION_PREDOMINANT = {
    **{t: 0.45 for t in range(10, 24)},
    **{t: 0.35 for t in range(42, 46)},
}
CONSTRAINT_LOW = {t: 0.30 for t in range(N_STEPS)}

EXPOSURE_CONSTRAINT_RESTORED = {
    **{t: 0.45 for t in range(10, 24)},
    **{t: 0.35 for t in range(42, 46)},
}
CONSTRAINT_RESTORED = {t: (0.30 if t < 30 else 0.58) for t in range(N_STEPS)}
INTERVENTION_RESTORED = {t: 0.35 for t in range(30, 42)}

CONFIGS = {
    "S1_balanced": dict(
        A0=0.20,
        C0=0.65,
        T0=0.20,
        B0=0.00,
        exposure_schedule=EXPOSURE_BALANCED,
        constraint_shift=CONSTRAINT_BALANCED,
    ),
    "S2_activation_predominant": dict(
        A0=0.25,
        C0=0.30,
        T0=0.20,
        B0=0.00,
        exposure_schedule=EXPOSURE_ACTIVATION_PREDOMINANT,
        constraint_shift=CONSTRAINT_LOW,
    ),
    "S3_constraint_restored": dict(
        A0=0.25,
        C0=0.30,
        T0=0.20,
        B0=0.00,
        exposure_schedule=EXPOSURE_CONSTRAINT_RESTORED,
        constraint_shift=CONSTRAINT_RESTORED,
        intervention_schedule=INTERVENTION_RESTORED,
    ),
}

CASE_LABELS = {
    "S1_balanced": "S1: Balanced",
    "S2_activation_predominant": "S2: Activation-predominant",
    "S3_constraint_restored": "S3: Constraint-restored",
}

CASE_COLORS = {
    "S1_balanced": "#2E7D32",
    "S2_activation_predominant": "#C62828",
    "S3_constraint_restored": "#1565C0",
}


# -----------------------------------------------------------------------------
# Monte Carlo sweep and summary
# -----------------------------------------------------------------------------

def run_monte_carlo(n_runs=300, n_steps=N_STEPS, seed_offset=1000):
    """
    Run each configuration n_runs times with independent random seeds.

    Returns a dictionary of stacked arrays with shape (n_runs, n_steps), per
    case and per variable. These arrays underlie the reported mean +/- SD
    trajectories and ordering-robustness checks.
    """
    all_results = {}

    for label, cfg in CONFIGS.items():
        A_runs = np.zeros((n_runs, n_steps))
        C_runs = np.zeros((n_runs, n_steps))
        T_runs = np.zeros((n_runs, n_steps))
        B_runs = np.zeros((n_runs, n_steps))

        for run in range(n_runs):
            A, C, T, B, I = simulate_case(
                seed=seed_offset + run,
                n_steps=n_steps,
                **cfg,
            )
            A_runs[run] = A
            C_runs[run] = C
            T_runs[run] = T
            B_runs[run] = B

        all_results[label] = dict(A=A_runs, C=C_runs, T=T_runs, B=B_runs)

    return all_results


def summarize_ordering(results):
    """
    Report how often the predicted ordering S1 < S3 < S2 holds for peak Burden,
    peak Tissue Context shift, and peak Activation during the second exposure.
    """
    n_runs = results["S1_balanced"]["A"].shape[0]
    window = slice(42, 51)

    peak_b = {k: v["B"].max(axis=1) for k, v in results.items()}
    peak_t = {k: v["T"].max(axis=1) for k, v in results.items()}
    peak_a2 = {k: v["A"][:, window].max(axis=1) for k, v in results.items()}

    def ordering_count(metric):
        s1 = metric["S1_balanced"]
        s2 = metric["S2_activation_predominant"]
        s3 = metric["S3_constraint_restored"]
        return int(np.sum((s1 < s3) & (s3 < s2))), n_runs

    print(f"Monte Carlo ordering check (S1 < S3 < S2), N={n_runs} runs:")
    for name, metric in [
        ("Peak Burden", peak_b),
        ("Peak Tissue shift", peak_t),
        ("Peak Activation (2nd exposure)", peak_a2),
    ]:
        holds, total = ordering_count(metric)
        print(f"  {name}: holds in {holds}/{total} runs ({100 * holds / total:.1f}%)")

    print(f"\n{'case':<28}{'peakB mean+/-sd':>18}{'peakT mean+/-sd':>18}{'peakA2 mean+/-sd':>18}")
    for label in CONFIGS:
        print(
            f"{label:<28}"
            + f"{peak_b[label].mean():.3f}+/-{peak_b[label].std():.3f}".rjust(18)
            + f"{peak_t[label].mean():.3f}+/-{peak_t[label].std():.3f}".rjust(18)
            + f"{peak_a2[label].mean():.3f}+/-{peak_a2[label].std():.3f}".rjust(18)
        )

    all_finite = all(
        np.all(np.isfinite(v["A"]))
        and np.all(np.isfinite(v["B"]))
        and np.all(np.isfinite(v["T"]))
        and np.all((v["A"] >= 0) & (v["A"] <= 1))
        and np.all((v["B"] >= 0) & (v["B"] <= 1))
        and np.all((v["T"] >= 0) & (v["T"] <= 1))
        for v in results.values()
    )
    print(f"\nAll {n_runs * len(CONFIGS)} simulated trajectories finite and bounded: {all_finite}")


# -----------------------------------------------------------------------------
# Figures
# -----------------------------------------------------------------------------

def make_figure(results, n_steps=N_STEPS, outpath="act_toy_figure.png"):
    """Create the four-panel mean +/- SD trajectory figure."""
    fig, axes = plt.subplots(2, 2, figsize=(11, 8))
    panels = [
        ("A", "Activation A(t)"),
        ("C", "Constraint C(t)"),
        ("B", "Latent Burden B(t) [not an ACT axis]"),
        ("T", "Tissue Context T(t)"),
    ]
    time = np.arange(n_steps)

    for ax, (var, title) in zip(axes.flat, panels):
        for label, res in results.items():
            data = res[var]
            mean = data.mean(axis=0)
            sd = data.std(axis=0)
            ax.plot(time, mean, label=CASE_LABELS[label], color=CASE_COLORS[label], linewidth=2)
            ax.fill_between(time, mean - sd, mean + sd, color=CASE_COLORS[label], alpha=0.15)
        ax.set_title(title)
        ax.set_xlabel("time step")
        ax.set_ylabel(var)
        ax.set_ylim(-0.05, 1.05)
        ax.axhline(0, color="grey", linewidth=0.5)
        ax.axhline(1, color="grey", linewidth=0.5)

    axes[0, 0].legend(loc="upper left", fontsize=9)
    fig.suptitle("ACT Toy Model: Mean +/- SD Across 300 Monte Carlo Runs per Configuration", fontsize=12)
    fig.tight_layout(rect=[0, 0, 1, 0.96])
    fig.savefig(outpath, dpi=200)
    print(f"\nFigure saved to {outpath}")


def ternary_project(A, C, T, eps=1e-9):
    """Project ACT coordinates to a ternary plot."""
    total = A + C + T + eps
    a = A / total
    c = C / total
    t = T / total
    x = c + 0.5 * t
    y = (np.sqrt(3) / 2.0) * t
    return x, y, a, c, t


def make_act_triangle_figure(results, n_steps=N_STEPS, outpath="act_triangle_trajectory.png"):
    """Create a ternary ACT trajectory figure with latent Burden overlay."""
    fig, axes = plt.subplots(1, 2, figsize=(12, 5), gridspec_kw={"width_ratios": [1.1, 1.0]})
    ax_tri, ax_burden = axes

    tri_x = [0, 1, 0.5, 0]
    tri_y = [0, 0, np.sqrt(3) / 2.0, 0]
    ax_tri.plot(tri_x, tri_y, color="black", linewidth=1)
    ax_tri.text(-0.04, -0.04, "Activation", ha="right", va="top", fontsize=10)
    ax_tri.text(1.04, -0.04, "Constraint", ha="left", va="top", fontsize=10)
    ax_tri.text(0.5, np.sqrt(3) / 2.0 + 0.04, "Tissue Context", ha="center", va="bottom", fontsize=10)

    time = np.arange(n_steps)
    for label, res in results.items():
        A_mean = res["A"].mean(axis=0)
        C_mean = res["C"].mean(axis=0)
        T_mean = res["T"].mean(axis=0)
        B_mean = res["B"].mean(axis=0)
        B_sd = res["B"].std(axis=0)

        x, y, _, _, _ = ternary_project(A_mean, C_mean, T_mean)
        ax_tri.plot(x, y, color=CASE_COLORS[label], linewidth=2, label=CASE_LABELS[label])
        marker_sizes = 20 + 180 * B_mean
        ax_tri.scatter(x[::4], y[::4], s=marker_sizes[::4], color=CASE_COLORS[label], alpha=0.45,
                       edgecolor="black", linewidth=0.3)
        ax_tri.scatter(x[0], y[0], color=CASE_COLORS[label], marker="o", s=45, edgecolor="black")
        ax_tri.scatter(x[-1], y[-1], color=CASE_COLORS[label], marker="X", s=70, edgecolor="black")

        ax_burden.plot(time, B_mean, color=CASE_COLORS[label], linewidth=2, label=CASE_LABELS[label])
        ax_burden.fill_between(time, np.maximum(0, B_mean - B_sd), np.minimum(1, B_mean + B_sd),
                               color=CASE_COLORS[label], alpha=0.15)

    ax_tri.set_title("ACT trajectory space with latent Burden overlay")
    ax_tri.set_aspect("equal")
    ax_tri.axis("off")
    ax_tri.legend(loc="lower center", bbox_to_anchor=(0.5, -0.18), ncol=1, fontsize=9)

    ax_burden.set_title("Latent Burden reference trace")
    ax_burden.set_xlabel("time step")
    ax_burden.set_ylabel("B(t), latent")
    ax_burden.set_ylim(-0.05, 1.05)
    ax_burden.axhline(0, color="grey", linewidth=0.5)
    ax_burden.axhline(1, color="grey", linewidth=0.5)
    ax_burden.legend(loc="upper left", fontsize=9)

    fig.suptitle("ACT Toy Model: Trajectory Through Reduced Coordinate Space", fontsize=12)
    fig.tight_layout()
    fig.savefig(outpath, dpi=200)
    print(f"\nACT triangle trajectory figure saved to {outpath}")


# -----------------------------------------------------------------------------
# Main
# -----------------------------------------------------------------------------

if __name__ == "__main__":
    results = run_monte_carlo(n_runs=300)
    summarize_ordering(results)
    make_figure(results)
    make_act_triangle_figure(results)
```

# S5. Code and Data Availability

All code, parameter configurations, and random seeds required to reproduce the results reported in Section 6.10. No patient data were used in this demonstration; all data shown are synthetic and generated by the code provided.
